# Morpho-phylogenetic evidence reveals five novel species of *Pestalotiopsis* (Sporocadaceae, Amphisphaeriales) from southern China

**DOI:** 10.3897/mycokeys.128.181974

**Published:** 2026-02-11

**Authors:** Ming-Gen Liao, Fang-Hua Guo, Xing-Xing Luo, Lian-Hu Zhang, Ru-Qiang Cui, Zhao-Huan Xu, Xiu-Guo Zhang, Jian Ma

**Affiliations:** 1 College of Agronomy, Jiangxi Agricultural University, Nanchang, Jiangxi 330045, China College of Agronomy, Jiangxi Agricultural University Nanchang China; 2 College of Plant Protection, Shandong Agricultural University, Taian, Shandong, 271018, China Jiangxi Key Laboratory for Excavation and Utilization of Agricultural Microorganisms, Jiangxi Agricultural University Nanchang China; 3 Jiangxi Key Laboratory for Excavation and Utilization of Agricultural Microorganisms, Jiangxi Agricultural University, Nanchang, Jiangxi 330045, China College of Plant Protection, Shandong Agricultural University Taian China

**Keywords:** Ascomycota, molecular phylogeny, new species, Sordariomycetes, taxonomy

## Abstract

Pestalotioid fungi occurring as plant pathogens, endophytes, or saprophytes exhibit a wide range of plant hosts. During our ongoing mycological surveys in southern China, 21 strains of *Pestalotiopsis* were isolated from diseased leaves of terrestrial plants. Phylogenetic analyses of ITS, *tef*1-α, and *tub*2 sequence data were performed using maximum likelihood and Bayesian inference to reveal their taxonomic placement within *Pestalotiopsis*. Both molecular phylogenetic analyses and morphological comparisons supported them as five new species of *Pestalotiopsis*, namely *P. acericola*, *P. corchorifolii*, *P. fraseri*, *P. goeppertiae*, and *P. koelreuteriae*, and one known species, *P. machiliana*. This study supplements the species diversity of *Pestalotiopsis* associated with multiple hosts in southern China.

## Introduction

Fungi represent one of the most diverse groups of organisms globally, playing critical roles in areas such as culinary value, ecological regulation, nutrient cycling, and innovative applications in biotechnology, medicine, and ecological conservation (Niego et al. 2023). Projections derived from host-association models estimate global fungal diversity at 2.2 to 3.8 million species, whereas high-throughput sequencing approaches yield substantially higher estimates in the range of 11.7 to 13.2 million species (Hawksworth and Lücking 2017; Hyde 2022). However, only around 200,000 fungal species have been discovered and formally documented in Fungal Names (FN, https://nmdc.cn/fungalnames/).

The genus *Pestalotiopsis* Steyaert was segregated from *Pestalotia* De Not. by Steyaert (1949) to accommodate those taxa with 5-celled conidia based on conidial forms, and *Pestalotiopsis maculans* (Corda) Nag Raj (≡ *Sporocadus maculans* Corda) was designated as the type species (Nag Raj 1985; Maharachchikumbura et al. 2014; Wijayawardene et al. 2017). *Pestalotiopsis* species are easily characterized by fusiform or subcylindrical, 5-celled conidia with three pigmented median cells and the presence of apical and basal appendages (Ariyawansa and Hyde 2018; Yin et al. 2024). Maharachchikumbura et al. (2014) further proposed two segregated anamorphic genera from *Pestalotiopsis*, namely *Neopestalotiopsis* Maharachch., K.D. Hyde & Crous and *Pseudopestalotiopsis* Maharachch., K.D. Hyde & Crous, based on combined datasets (ITS, *tef*1-α, and *tub*2) and the conidial pigmentation of the three median cells. As a result, *Neopestalotiopsis* differs from *Pestalotiopsis* and *Pseudopestalotiopsis* by its conidia with versicolorous median cells, and *Pseudopestalotiopsis* can be distinguished from *Pestalotiopsis* by sequence data and its conidia with dark-colored concolorous median cells. *Pestalotiopsis* is a species-rich group with varied habitats distributed in tropical and temperate regions (Bate-Smith and Metcalfe 1957; Luo et al. 2024). To date, about 457 epithets of *Pestalotiopsis* are recorded in Index Fungorum (Index Fungorum 2025). Members of the genus are common phytopathogens causing a variety of plant diseases, also occurring as endophytes and saprophytes isolated from leaf tissues, soil substrates, and rotted leaves, with some species further implicated in human and animal infections (Zhang et al. 2012a; Monden et al. 2013; Li et al. 2024; Luo et al. 2024; Yin et al. 2024). Previous research on *Pestalotiopsis* across a spectrum of environments has revealed a range of ecological functions. For example, functioning as saprophytes, some species accelerate material decomposition, whereas as endophytes, some species serve as sources of novel compounds with medicinal, agricultural, and industrial relevance (Xu et al. 2010, 2014).

Maharachchikumbura et al. (2014) revised the genus *Pestalotiopsis* based on combined ITS, *tef*1-α, and *tub*2 sequence data and morphological characteristics. These loci demonstrated strong phylogenetic resolution at the genus level and have been widely adopted in subsequent phylogenetic studies (Lin et al. 2023; Sun et al. 2023; Wang et al. 2025). In this study, samples of diseased leaves were collected from southern China. Accordingly, based on morphological observations and phylogenetic analyses (ITS, *tef*1-α, and *tub*2), 21 strains were assigned to *Pestalotiopsis*, including five novel species, namely *P. acericola*, *P. corchorifolii*, *P. fraseri*, *P. goeppertiae*, and *P. koelreuteriae*, and one known species, *P. machiliana* X.X. Luo & Jian Ma. Detailed illustrations and morphological descriptions of these taxa are provided below.

## Materials and methods

### Sample collection, fungal isolation, and morphological observation

Samples of diseased plant leaves were collected from botanical gardens and forest parks in southern China. The samples were placed in sealed bags with filter paper and transported to the laboratory. Fungi were isolated according to the tissue isolation method described by Gao et al. (2014). Leaf tissues were transferred onto potato dextrose agar (PDA; 200 g potato, 20 g glucose, 20 g agar, and 1000 mL water) plates and cultured at 25 °C under alternating light and dark conditions (8 h/16 h) for seven days, after which cultural characteristics, including color, shape, and size, were observed and recorded. The microscopic structures of isolated fungi were examined using an Olympus BX53 light microscope equipped with an Olympus DP27 digital camera (Olympus Optical Co., Ltd., Tokyo, Japan) to describe their morphological characteristics. All fungal strains were stored in 10% sterilized glycerin at 4 °C for further studies. The studied specimens and cultures were deposited in the Herbarium of Jiangxi Agricultural University, Plant Pathology, Nanchang, China (**HJAUP**).

### DNA extraction, PCR amplification, and sequencing

After fungal cultures were grown at 25 °C under a light–dark cycle (8 h/16 h) for 14 days, fresh mycelia were scraped with a sterile needle and transferred into 2 mL centrifuge tubes for grinding with liquid nitrogen. Genomic DNA was extracted using the Solarbio Fungal Genomic DNA Extraction Kit (Beijing Solarbio Science & Technology Co., Ltd., Beijing, China) according to the manufacturer’s protocol. DNA was amplified in a 25 μL PCR system consisting of 12.5 µL of 2 × Power Taq PCR MasterMix, 9.5 µL of double-distilled water (ddH_2_O), 1 µL of DNA template, and 1 µL each of forward and reverse primers, targeting three loci: ITS, *tef*1-α, and *tub*2. The primer pairs and PCR programs are listed in Table 1. PCR amplification products were stained with ethidium bromide and examined by electrophoresis in 1% agarose gels. Purification and sequencing of the PCR products were performed by Hunan Youkanglai Biotechnology Co., Ltd. The obtained sequences were submitted to the GenBank database (www.ncbi.nlm.nih.gov, accessed on 1 December 2025; Table 2).

**Table 1.** Primers and PCR program used in this study.

Locus	Primers		PCR program
	Name	Sequence 5'–3'	
ITS	ITS5	GGAAGTAAAAGTCGTAACAAGG	95 °C: 3 min, (95 °C: 15 s, 54 °C: 15 s, 72 °C: 30 s) × 35 cycles, 72 °C: 5 min
	ITS4	TCCTCCGCTTATTGATATGC	
*tef*1*-α*	EF1-728F	CATCGAGAAGTTCGAGAAGG	95 °C: 3 min, (95 °C: 15 s, 56 °C: 15 s, 72 °C: 30 s) × 35 cycles, 72 °C: 5 min
	EF1-986R	TACTTGAAGGAACCCTTACC	
*tub*2	Bt2a	GGTAACCAAATCGGTGCTGCTTTC	95 °C: 3 min, (95 °C: 15 s, 54 °C: 15 s, 72 °C: 30 s) × 35 cycles, 72 °C: 5 min
	Bt2b	ACCCTCAGTGTAGTGACCCTTGGC	

**Table 2.** *Pestalotiopsis* species with their GenBank accession numbers used in the phylogenetic analyses of this study. New sequences are in bold.

Species	Strain number	Host/substrate	Locality	GenBank accession number			References
				ITS	*tef*1-*α*	*tub*2	
*Pestalotiopsis abietis*	CFCC 53011^T^	*Abies fargesii*	China	MK397013	MK622277	MK622280	Gu et al. (2021)
*P. abietis*	CFCC 53012	*Abies fargesii*	China	MK397014	MK622278	MK622281	
*P. adusta*	ICMP 6088^T^	Refrigerator door	Fiji	JX399006	JX399070	JX399037	Maharachchikumbura et al. (2012)
***P. acericola***	**HJAUP C1856.1^T^**	***Acer buergerianum***	**China**	**PX613543**	**PX617762**	**PX625971**	**This study**
***P. acericola***	**HJAUP C1856.2**	***Acer buergerianum***	**China**	**PX613544**	**PX617763**	**PX625972**	
***P. acericola***	**HJAUP C1856.3**	***Acer buergerianum***	**China**	**PX613545**	**PX617764**	**PX625973**	
***P. acericola***	**HJAUP C1856.4**	***Acer buergerianum***	**China**	**PX613546**	**PX617765**	**PX625974**	
*P. aggestorum*	LC 6301^T^	*Camellia sinensis*	China	KX895015	KX895234	KX895348	Liu et al. (2017)
*P. aggestorum*	LC 8186	*Camellia sinensis*	China	KY464140	KY464150	KY464160	
*P. alloschemones*	CGMCC 3.23480^T^	*Alloschemone occidentalis*	China	OR247981	OR361456	OR381056	Razaghi et al. (2024)
*P. alpinicola*	HJAUP C1644.221^T^	*Alpinia zerumbet*	China	PP962274	PP952249	PP952219	Luo et al. (2024)
*P. alpinicola*	HJAUP C1644.222	*Alpinia zerumbet*	China	PP962275	PP952248	PP952220	
*P. americana*	CBS 111576^T^	*Leucospermum cunei* × *conocarpodendron*	USA	MH553961	MH554379	MH554620	Liu et al. (2019)
*P. anacardiacearum*	IFRDCC 2397^T^	*Mangifera indica*	China	KC247154	KC247156	KC247155	Maharachchikumbura et al. (2013a)
*P. anhuiensis*	CFCC 54791^T^	*Cyclobalanopsis glauca*	China	ON007028	ON005045	ON005056	Jiang et al. (2022a)
*P. aporosae-dioicae*	SAUCC224004^T^	*Aporosa dioica*	China	OR733506	OR912988	OR912985	Yin et al. (2024)
*P. aporosae-dioicae*	SAUCC224005	*Aporosa dioica*	China	OR733505	OR912989	OR912986	
*P. appendiculata*	CGMCC 3.23550^T^	*Rhododendron decorum*	China	OP082431	OP185509	OP185516	Gu et al. (2022)
*P. arceuthobii*	CBS 434.65^T^	*Arceuthobium campylopodum*	USA	KM199341	KM199516	KM199427	Maharachchikumbura et al. (2014)
*P. arengae*	CBS 331.92^T^	*Arenga undulatifolia*	Singapore	KM199340	KM199515	KM199426	Maharachchikumbura et al. (2014)
*P. australasiae*	CBS 114126^T^	*Knightia* sp.	New Zealand	KM199297	KM199499	KM199409	Maharachchikumbura et al. (2014)
*P. australasiae*	CBS 114141	*Protea* sp.	New South Wales	KM199298	KM199501	KM199410	
*P. australis*	CBS 111503	*Protea neriifolia* × *susannae* cv. ‘Pink Ice’	South Africa	KM199331	KM199557	KM199382	Maharachchikumbura et al. (2014)
*P. australis*	CBS 114193^T^	*Grevillea* sp.	New South Wales	KM199332	KM199475	KM199383	
*P. biappendiculata*	CGMCC 3.23487^T^	*Rhododendron* sp.	China	OR247984	OR361459	OR381059	Razaghi et al. (2024)
*P. biappendiculata*	LC4282	*Rhododendron* sp.	China	OR247990	OR361465	OR381065	
*P. biappendiculata*	LC4283	*Rhododendron* sp.	China	OR247991	OR361466	OR381066	
*P. biciliata*	CBS 124463^T^	*Platanus × hispanica*	Slovakia	KM199308	KM199505	KM199399	Maharachchikumbura et al. (2014)
*P. biciliata*	CBS 236.38	*Paeonia* sp.	Italy	KM199309	KM199506	KM199401	
*P. brachiata*	LC 2988^T^	*Camellia* sp.	China	KX894933	KX895150	KX895265	Liu et al. (2017)
*P. brachiata*	LC 8188	*Camellia* sp.	China	KY464142	KY464152	KY464162	
*P. brachiata*	LC 8189	*Camellia* sp.	China	KY464143	KY464153	KY464163	
*P. brassicae*	CBS 170.26^T^	*Brassica napus*	New Zealand	KM199379	KM199558	–	Maharachchikumbura et al. (2014)
*P. buxicola*	CFCC 57357^T^	*Buxus bodinieri*	China	PP965518	PP957899	PP957901	Jiang et al. (2025)
*P. buxicola*	CFCC 57358	*Buxus bodinieri*	China	PP965519	PP957900	PP957902	
*P. camelliicola*	HJAUP C1804.22^1^T	*Camellia japonica*	China	PP962357	PP952236	PP952229	Luo et al. (2024)
*P. camelliicola*	HJAUP C1804.222	*Camellia japonica*	China	PP962358	PP952235	PP952230	
*P. camelliae*	MFLUCC 12–0277^T^	*Camellia japonica*	China	JX399010	JX399074	JX399041	Zhang et al. (2012a)
*P. camelliae–oleiferae*	CSUFTCC 08^T^	*Camelliae oleiferae*	China	OK493593	OK507963	OK562368	Li et al. (2021)
*P. camelliae–oleiferae*	CSUFTCC 09	*Camelliae oleiferae*	China	OK493594	OK507964	OK562369	
*P. cangshanensis*	CGMCC 3.23544^T^	*Rhododendron delavayi*	China	OP082426	OP185510	OP185517	Gu et al. (2022)
*P. castanopsidis*	CFCC 54430^T^	*Castanopsis lamontii*	China	OK339732	OK358493	OK358508	Jiang et al. (2022a)
*P. castanopsidis*	CFCC 54305	*Castanopsis hystrix*	China	OK339733	OK358494	OK358509	
*P. castanopsidis*	CFCC 54384	*Castanopsis hystrix*	China	OK339734	OK358495	OK358510	
*P. chamaeropis*	CBS 186.71^T^	*Chamaerops humilis*	Italy	KM199326	KM199473	KM199391	Maharachchikumbura et al. (2014)
*P. chamaeropis*	CFCC 55122	*Quercus aliena*	China	OM746229	OM840001	OM839902	Jiang et al. (2022b)
*P. chamaeropis*	CFCC 55023	*Castanopsis fissa*	China	OM746233	OM840005	OM839906	
*P. changjiangensis*	CFCC 54314^T^	*Castanopsis tonkinensis*	China	OK339739	OK358500	OK358515	Jiang et al. (2022a)
*P. changjiangensis*	CFCC 54433	*Castanopsis hainanensis*	China	OK339740	OK358501	OK358516	
*P. changjiangensis*	CFCC 52803	*Cyclobalanopsis austrocochinchinensis*	China	OK339741	OK358502	OK358517	
*P. chaoyangensis*	CFCC 55549^T^	*Euonymus japonicus*	China	OQ344763	OQ410582	OQ410584	Lin et al. (2023)
*P. chaoyangensis*	CFCC 58805	*Euonymus japonicus*	China	OQ344764	OQ410583	OQ410585	
*P. chiangmaiensis*	MFLUCC 22–0127^T^	*Phyllostachys edulis*	Thailand	OP497990	OP753374	OP752137	Sun et al. (2023)
*P. chiaroscuro*	BRIP 72970^T^	*Sporobolus natalensis*	Australia	OK422510	OK423753	OK423752	Crous et al. (2022)
*P. chinensis*	MFLUCC 12–0273^T^	NA	China	JX398995	–	–	Maharachchikumbura et al. (2012)
*P. clavata*	MFLUCC 12–0268^T^	*Buxus* sp.	China	JX398990	JX399056	JX399025	Maharachchikumbura et al. (2012)
*P. colombiensis*	CBS 118553^T^	*Eucalyptus urograndis*	Colombia	KM199307	KM199488	KM199421	Maharachchikumbura et al. (2014)
***P. corchorifolii***	**HJAUP C1891.1^T^**	***Rubus corchorifolius***	**China**	**PX613547**	**PX617766**	**PX625975**	**This study**
***P. corchorifolii***	**HJAUP C1891.2**	***Rubus corchorifolius***	**China**	**PX613548**	**PX617767**	**PX625976**	
***P. corchorifolii***	**HJAUP C1891.3**	***Rubus corchorifolius***	**China**	**PX613549**	**PX617768**	**PX625977**	
***P. corchorifolii***	**HJAUP C1891.4**	***Rubus corchorifolius***	**China**	**PX613550**	**PX617769**	**PX625978**	
***P. corchorifolii***	**HJAUP C1891.5**	***Rubus corchorifolius***	**China**	**PX613551**	**PX617770**	**PX625979**	
*P. cratoxyli*	CGMCC 3.23512^T^	*Cratoxylum cochinchinense*	China	OR248005	OR361480	OR381080	Razaghi et al. (2024)
*P. cratoxyli*	LC8772	*Cratoxylum cochinchinense*	China	OR248004	OR361479	OR381079	
*P. cyclobalanopsidis*	CFCC 54328^T^	*Cyclobalanopsis glauca*	China	OK339735	OK358496	OK358511	Jiang et al. (2022a)
*P. cyclobalanopsidis*	CFCC 55891	*Cyclobalanopsis glauca*	China	OK339736	OK358497	OK358512	
*P. cyclosora*	HJAUP C1724.221^T^	*Cyclosorus interruptus*	China	PP962279	PP952247	PP952221	Luo et al. (2024)
*P. cyclosora*	HJAUP C1724.222	*Cyclosorus interruptus*	China	PP962280	PP952246	PP952222	
*P. cyclosora*	HJAUP C1725.221	*Microlepia marginata*	China	PP962281	PP952245	PP952223	
*P. cyclosora*	HJAUP C1725.222	*Microlepia marginata*	China	PP962282	PP952244	PP952233	
*P. cyclosora*	HJAUP C1726.221	*Punica granatum*	China	PP962283	PP952243	PP952224	
*P. cyclosora*	HJAUP C1726.222	*Punica granatum*	China	PP962284	PP952242	PP952232	
*P. daliensis*	CGMCC 3.23548^T^	*Rhododendron decorum*	China	OP082429	OP185511	OP185518	Gu et al. (2022)
*P. dianellae*	CBS 143421^T^	*Dianella* sp.	Australia	MG386051	–	MG386164	Crous et al. (2017)
*P. digitalis*	MFLU 14–0208^T^	*Digitalis purpurea*	New Zealand	KP781879	–	KP781883	Liu et al. (2015)
*P. dilucida*	LC3232^T^	*Camellia sinensis*	China	KX894961	KX895178	KX895293	Liu et al. (2017)
*P. dilucida*	LC8184	*Camellia sinensis*	China	KY464138	KY464148	KY464158	
*P. diploclisiae*	CBS 115587^T^	*Diploclisia glaucescens*	China	KM199320	KM199486	KM199419	Maharachchikumbura et al. (2014)
*P. disseminata*	CBS 143904	*Persea americana*	New Zealand	MH554152	MH554587	MH554825	Liu et al. (2019)
*P. disseminata*	MEAN 1165	*Pinus pinea*	Portugal	MT374687	MT374699	MT374712	Silva et al. (2020)
*P. diversiseta*	MFLUCC 12–0287^T^	*Rhododendron* sp.	China	JX399009	JX399073	JX399040	Maharachchikumbura et al. (2012)
*P. doitungensis*	MFLUCC 14–0115	*Dendrobium* sp.	Thailand	MK993574	MK975832	MK975837	Ma et al. (2019)
*P. dracaenae*	HGUP 4037^T^	*Dracaena fragrans*	China	–	MT598644	MT598645	Ariyawansa et al. (2015)
*P. dracaenicola*	MFLUCC 18–0913^T^	*Dracaena* sp.	Thailand	MN962731	MN962732	MN962733	Chaiwan et al. (2020)
*P. dracaenicola*	MFLUCC 18–0914	*Dracaena* sp.	Thailand	MN962734	MN962735	MN962736	
*P. dracontomelonis*	MFLU 14–0207	*Dracontomelon dao*	Thailand	KP781877	KP781880	–	Liu et al. (2015)
*P. eleuthero–cocci*	HMJAU 60189^T^	*Eleutherococcus brachypus*	China	OL996126	–	–	Tian et al. (2022)
*P. eleuthero–cocci*	HMJAU 60190	*Eleutherococcus brachypus*	China	OL996127	–	OL898722	
*P. endophytica*	MFLUCC 18–0932^T^	*Magnolia garrettii*	Thailand	MW263946	MW417119	–	de Silva et al. (2021)
*P. endophytica*	MFLUCC 18–0946	*Magnolia garrettii*	Thailand	MW263947	MW729384	–	
*P. ericacearum*	IFRDCC 2439^T^	*Rhododendron delavayi*	China	KC537807	KC537814	KC537821	Zhang et al. (2013)
*P. eriobotryae*	HJAUP C1742.221^T^	*Eriobotrya japonica*	China	PP962289	PP952238	PP952227	Luo et al. (2024)
*P. eriobotryae*	HJAUP C1742.222	*Eriobotrya japonica*	China	PP962291	PP952237	PP952228	
*P. etonensis*	BRIP 66615^T^	*Sporobolus jacquemontii*	Australia	MK966339	MK977635	MK977634	Crous et al. (2020)
*P. exudata*	CGMCC 3.23488^T^	*Aucuba japonica*	China	OR247985	OR361460	OR381060	Razaghi et al. (2024)
*P. exudata*	LC15850	*Aucuba japonica*	China	OR247986	OR361461	OR381061	
*P. ficicrescens*	HGUP 861^T^	*Camellia japonica*	China	MZ477311	MZ868328	MZ868301	Hyde et al. (2023)
*P. ficicrescens*	CGMCC 3.23471	Oleaceae	China	OR247980	OR361455	OR381055	Razaghi et al. (2024)
*P. foliicola*	CFCC 54440^T^	*Castanopsis faberi*	China	ON007029	ON005046	ON005057	Jiang et al. (2022a)
*P. foliicola*	CFCC 57359	*Castanopsis faberi*	China	ON007030	ON005047	ON005058	
*P. foliicola*	CFCC 57360	*Castanopsis faberi*	China	ON007031	ON005048	ON005059	
*P. formosana*	NTUCC 17–009^T^	Poaceae sp.	China	MH809381	MH809389	MH809385	Ariyawansa and Hyde (2018)
*P. formosana*	NTUCC 17–010	Poaceae sp.	China	MH809382	MH809390	MH809386	
***P. fraseri***	**HJAUP C1693.1^T^**	***Photinia × fraseri***	**China**	**PX613552**	**PX617771**	**PX625980**	**This study**
***P. fraseri***	**HJAUP C1693.2**	***Photinia × fraseri***	**China**	**PX613553**	**PX617772**	**PX625981**	
***P. fraseri***	**HJAUP C1693.3**	***Photinia × fraseri***	**China**	**PX613554**	**PX617773**	**PX625982**	
***P. fraseri***	**HJAUP C1693.4**	***Photinia × fraseri***	**China**	**PX613555**	**PX617774**	**PX625983**	
*P. furcata*	MFLUCC 12–0054^T^	*Camellia sinensis*	Thailand	JQ683724	JQ683740	JQ683708	Maharachchikumbura et al. (2013b)
*P. furcata*	LC6691	*Camellia sinensis*	China	KX895030	KX895248	KX895363	Liu et al. (2017)
*P. fusiformis*	CGMCC 3.23495^T^	*Rhododendron* sp.	China	OR247995	OR361470	OR381070	Razaghi et al. (2024)
*P. fusiformis*	LC15852	*Rhododendron* sp.	China	OR247996	OR361471	OR381071	
*P. fusoidea*	CGMCC 3.23545^T^	*Rhododendron delavayi*	China	OP082427	OP185512	OP185519	Gu et al. (2022)
*P. ganzhouensis*	CGMCC 3.23489^T^	*Cinnamomum camphora*	China	OR247987	OR361462	OR381062	Razaghi et al. (2024)
*P. ganzhouensis*	LC5089	*Cinnamomum camphora*	China	OR247998	OR361473	OR381073	
*P. gardeniae*	HJAUP C1729.221^T^	*Gardenia jasminoides*	China	PP962285	PP952241	PP952225	Luo et al. (2024)
*P. gardeniae*	HJAUP C1729.222	*Gardenia jasminoides*	China	PP962286	PP952240	PP952226	
*P. gardeniae*	HJAUP C1729.223	*Gardenia jasminoides*	China	PP962287	PP952239	PP952231	
*P. gaultheriae*	IFRD 411–014^T^	*Gaultheria forrestii*	China	KC537805	KC537812	KC537819	Zhang et al. (2013)
*P. gibbosa*	NOF 3175^T^	*Gaultheria shallon*	Canada	LC311589	LC311591	LC311590	Watanabe et al. (2018)
***P. goeppertiae***	**HJAUP C1919.1^T^**	***Goeppertia makoyana***	**China**	**PX613556**	**PX617775**	**PX625984**	**This study**
***P. goeppertiae***	**HJAUP C1919.2**	***Goeppertia makoyana***	**China**	**PX613557**	**PX617776**	**PX625985**	
***P. goeppertiae***	**HJAUP C1919.3**	***Goeppertia makoyana***	**China**	**PX613558**	**PX617777**	**PX625986**	
***P. goeppertiae***	**HJAUP C1919.4**	***Goeppertia makoyana***	**China**	**PX613559**	**PX617778**	**PX625987**	
*P. grevilleae*	CBS 114127^T^	*Grevillea* sp.	Australia	KM199300	KM199504	KM199407	Maharachchikumbura et al. (2014)
*P. guangdongensis*	ZHKUCC 22–0016^T^	*Arenga pinnata*	China	ON180762	ON221520	ON221548	Xiong et al. (2022)
*P. guangdongensis*	ZHKUCC 22–0017	*Arenga pinnata*	China	ON180763	ON221521	ON221549	
*P. guangdongensis*	ZHKUCC 22–0018	*Arenga pinnata*	China	ON180764	ON221522	ON221550	
*P. guangxiensis*	CFCC 54308^T^	*Quercus griffithii*	China	OK339737	OK358498	OK358513	Jiang et al. (2022a)
*P. guangxiensis*	CFCC 54300	*Quercus griffithii*	China	OK339738	OK358499	OK358514	
*P. guiyangensis*	CFCC 70626^T^	*Eriobotrya japonica*	China	PP784740	PP842629	PP842617	Zhang et al. (2024)
*P. guizhouensis*	CFCC 54803	*Cyclobalanopsis glauca*	China	ON007035	ON005052	ON005063	Jiang et al. (2022a)
*P. guizhouensis*	CFCC 57364^T^	*Cyclobalanopsis glauca*	China	ON007036	ON005053	ON005064	
*P. hawaiiensis*	CBS 114491^T^	*Leucospermum* sp.	USA	KM199339	KM199514	KM199428	Maharachchikumbura et al. (2014)
*P. hederae*	HJAUP C1638.221^T^	*Hedera helix*	China	PP962270	PP952252	PP952234	Luo et al. (2024)
*P. hederae*	HJAUP C1638.222	*Hedera helix*	China	PP962271	–	PP952216	
*P. hispanica*	CBS 115391^T^	*Protea* sp.	Spain	MH553981	MH554399	MH554640	Liu et al. (2019)
*P. hollandica*	CBS 265.33^T^	*Sciadopitys verticillata*	Netherlands	KM199328	KM199481	KM199388	Maharachchikumbura et al. (2014)
*P. hollandica*	MEAN 1091	*Pinus pinea*	Portugal	MT374678	MT374691	MT374703	Silva et al. (2020)
*P. humicola*	CBS 336.97^T^	Soil	Papua New Guinea	KM199317	KM199484	KM199420	Maharachchikumbura et al. (2014)
*P. hunanensis*	CSUFTCC15^T^	*Camellia oleifera*	China	OK493599	OK507969	OK562374	Li et al. (2021)
*P. hunanensis*	CSUFTCC18	*Camellia oleifera*	China	OK493600	OK507970	OK562375	
*P. hydei*	MFLUCC 20–0135^T^	*Litsea petiolata*	Thailand	MW266063	MW251113	MW251112	Huanaluek et al. (2021)
*P. iberica*	CAA 1004^T^	*Pinus radiata*	Spain	MW732248	MW759038	MW759035	Monteiro et al. (2022)
*P. iberica*	CAA 1006	*Pinus radiata*	Spain	MW732249	MW759039	MW759036	
*P. inflexa*	MFLUCC 12–0270^T^	Unidentified tree	China	JX399008	JX399072	JX399039	Maharachchikumbura et al. (2012)
*P. intermedia*	MFLUCC 12–0259^T^	Unidentified tree	China	JX398993	JX399059	JX399028	Maharachchikumbura et al. (2012)
*P. italiana*	MFLU 14–0214^T^	*Cupressus glabra*	Italy	KP781878	KP781881	KP781882	Liu et al. (2015)
*P. jesteri*	MFLUCC12–0279	*Fagraea bodenii*	China	JX399012	JX399076	JX399043	Maharachchikumbura et al. (2012)
*P. jiangmenensis*	CFCC 72595^T^	*Pinus massoniana*	China	PV259810	PV275131	PV275205	Wang et al. (2025)
*P. jiangmenensis*	CFCC 72596	*Pinus massoniana*	China	PV259811	PV275132	PV275206	
*P. jiangsuensis*	CFCC 59538	*Pinus massoniana*	China	OR533577	OR539186	OR539191	Li et al. (2024)
*P. jiangsuensis*	CFCC 59539	*Pinus massoniana*	China	OR533578	OR539187	OR539192	
*P. jiangsuensis*	CFCC 59542	*Pinus massoniana*	China	OR533581	OR539190	OR539195	
*P. jiangxiensis*	LC4399^T^	*Camellia* sp.	China	KX895009	KX895227	KX895341	Liu et al. (2017)
*P. jinchanghensis*	LC6636^T^	*Camellia sinensis*	China	KX895028	KX895247	KX895361	Liu et al. (2017)
*P. jinchanghensis*	LC8190	*Camellia sinensis*	China	KY464144	KY464154	KY464164	
*P. kaki*	KNU–PT–1804^T^	*Diospyros kaki*	Korea	LC552953	LC553555	LC552954	Das et al. (2020)
*P. kandelicola*	NCYUCC 19–0355^T^	*Kandelia candel*	China	MT560723	MT563102	MT563100	Hyde et al. (2020)
*P. kenyana*	CBS 442.67^T^	*Coffea* sp.	Kenya	KM199302	KM199502	KM199395	Maharachchikumbura et al. (2014)
*P. kenyana*	LC6633	*Camellia sinensis*	China	KX895027	KX895246	KX895360	
*P. kenyana*	CFCC 54962	*Quercus aliena*	China	OM746237	OM840009	OM839910	Jiang et al. (2022a)
*P. kenyana*	CFCC 54805	*Cyclobalanopsis glauca*	China	OM746253	OM840025	OM839926	
*P. kenyana*	CFCC 55088	*Castanopsis fissa*	China	OM746254	OM840026	OM839927	
*P. knightiae*	CBS 111963	*Knightia* sp.	New Zealand	KM199311	KM199495	KM199406	Maharachchikumbura et al. (2014)
*P. knightiae*	CBS 114138^T^	*Knightia* sp.	New Zealand	KM199310	KM199497	KM199408	
***P. koelreuteriae***	**HJAUP C2757.1^T^**	***Koelreuteria paniculata***	**China**	**PX613560**	**PX617779**	**PX625988**	**This study**
***P. koelreuteriae***	**HJAUP C2757.2**	***Koelreuteria paniculata***	**China**	**PX613561**	**PX617780**	**PX625989**	
*P. krabiensis*	MFLUCC 16–0260^T^	*Pandanus* sp.	Thailand	MH388360	MH388395	MH412722	Tibpromma et al. (2018)
*P. leucadendri*	CBS 121417^T^	*Leucadendron* sp.	South Africa	MH553987	MH554412	MH554654	Liu et al. (2019)
*P. leucospermi*	CBS 114489^T^	* Leucospermum *	USA	MH553978	MH554396	MH554637	Liu et al. (2019)
*P. licualicola*	HGUP 4057^T^	*Licuala grandis*	China	KC492509	KC481684	KC481683	Geng et al. (2013)
*P. lijiangensis*	CFCC 50738^T^	*Castanopsis carlesii* var. *spinulosa*	China	KU860520	KU844185	KU844184	Zhou et al. (2018)
*P. linearis*	MFLUCC 12–0271^T^	*Trachelospermum* sp.	China	JX398992	JX399058	JX399027	Maharachchikumbura et al. (2012)
*P. linguae*	ZHKUCC 22–0159^T^	*Pyrrosia lingua*	China	OP094104	OP186110	OP186108	Li et al. (2023)
*P. linguae*	ZHKUCC 22–0160	*Pyrrosia lingua*	China	OP094103	OP186109	OP186107	
*P. lithocarpi*	CFCC 55100^T^	*Lithocarpus chiungchungensis*	China	OK339742	OK358503	OK358518	Jiang et al. (2022a)
*P. lithocarpi*	CFCC 55893	*Lithocarpus chiungchungensis*	China	OK339743	OK358504	OK358519	
*P. lobata*	CGMCC 3.23467^T^	*Lithocarpus glaber*	China	OR247976	OR361451	OR381051	Razaghi et al. (2024)
*P. lobata*	LC15843	*Lithocarpus glaber*	China	OR247977	OR361452	OR381052	
*P. loeiana*	MFLUCC 22–0123^T^	Dead leaves	Thailand	OP497988	OP737881	OP713769	Sun et al. (2023)
*P. longiappendiculata*	LC3013	*Camellia sinensis*	China	KX894939	KX895156	KX895271	Liu et al. (2017)
*P. lushanensis*	LC4344^T^	*Camellia* sp.	China	KX895005	KX895223	KX895337	Liu et al. (2017)
*P. lushanensis*	LC8182	*Camellia* sp.	China	KY464136	KY464146	KY464156	
*P. lushanensis*	LC8183	*Camellia* sp.	China	KY464137	KY464147	KY464157	
*P. lushanensis*	CFCC 54894	*Quercus serrata*	China	OM746282	OM840054	OM839955	Jiang et al. (2022b)
*P. macadamiae*	BRIP 63738b^T^	*Macadamia integrifolia*	Australia	KX186588	KX186621	KX186680	Akinsanmi et al. (2017)
*P. macadamiae*	BRIP 63739b	*Macadamia integrifolia*	Australia	KX186587	KX186620	KX186679	
*P. macadamiae*	BRIP 637441a	*Macadamia integrifolia*	Australia	KX186586	KX186619	KX186678	
*P. machili*	CGMCC 3.23511^T^	*Machilus* sp.	China	OR248003	OR361478	OR381078	Razaghi et al. (2024)
*P. machiliana*	HJAUP C1790.221^T^	*Machilus pauhoi*	China	PP962355	PP952253	PP952214	Luo et al. (2024)
*P. machiliana*	HJAUP C1790.222	*Machilus pauhoi*	China	PP962356	PP952254	PP952215	
*P. machiliana*	HJAUP C1704.221	*Rhododendron simsii*	China	PP962276	PP952255	PP952211	
*P. machiliana*	HJAUP C1704.222	*Rhododendron simsii*	China	PP962277	PP952256	PP952212	
*P. machiliana*	HJAUP C1704.223	*Rhododendron simsii*	China	PP962278	PP952257	PP952213	
***P. machiliana***	**HJAUP C2801.1**	***Bambusa multiplex***	**China**	**PX613562**	**PX617781**	**PX625990**	**This study**
***P. machiliana***	**HJAUP C2801.2**	***Bambusa multiplex***	**China**	**PX613563**	**PX617782**	**PX625991**	
*P. malayana*	CBS 102220^T^	*Macaranga triloba*	Malaysia	KM199306	KM199482	KM199411	Maharachchikumbura et al. (2014)
*P. mangifericola*	HJAUP C1639.221^T^	*Mangifera indica*	China	PP962272	PP952251	PP952217	Luo et al. (2024)
*P. mangifericola*	HJAUP C1639.222	*Mangifera indica*	China	PP962273	PP952250	PP952218	
*P. manyueyuanensis*	NTUPPMCC 18-165^T^	*Ophiocordyceps* sp.	China	OR125060	OR126313	OR126306	Hsu et al. (2024)
*P. manyueyuanensis*	NTUPPMCC 22-012	*Ophiocordyceps* sp.	China	OR125061	OR126314	OR126307	
*P. massoniana*	CFCC 72593^T^	*Pinus massoniana*	China	PV259820	PV275141	PV275215	Wang et al. (2025)
*P. massoniana*	CFCC 72594	*Pinus massoniana*	China	PV259821	PV275142	PV275216	
*P. menhaiensis*	YN3A1^T^	*Camellia sinensis*	China	KU252272	KU252401	KU252488	Wang et al. (2019)
*P. monochaeta*	CBS 144.97^T^	*Quercus robur*	Netherlands	KM199327	KM199479	KM199386	Maharachchikumbura et al. (2014)
*P. monochaeta*	CBS 440.83	*Taxus baccata*	Netherlands	KM199329	KM199480	KM199387	
*P. multiappendiculata*	CGMCC 3.23514^T^	NA	China	OR248008	OR361483	OR381083	Razaghi et al. (2024)
*P. multicolor*	CFCC59981^T^	*Taxus chinensis*	China	OQ626676	OQ714341	OQ714336	Wang et al. (2024)
*P. multicolor*	CFCC59982	*Taxus chinensis*	China	OQ771896	OQ779483	OQ779488	
*P. nanjingensis*	CSUFTCC20	*Camellia oleifera*	China	OK493603	OK507973	OK562378	Li et al. (2021)
*P. nanjingensis*	CSUFTCC04	*Camellia oleifera*	China	OK493604	OK507974	OK562379	
*P. nanningensis*	CSUFTCC10^T^	*Camellia oleifera*	China	OK493596	OK507966	OK562371	Li et al. (2021)
*P. nanningensis*	CSUFTCC11	*Camellia oleifera*	China	OK493597	OK507967	OK562372	
*P. nannuoensis*	SAUCC232203^T^	Unknown host	China	OR733504	OR912991	OR863909	Yin et al. (2024)
*P. nannuoensis*	SAUCC232204	Unknown host	China	OR733503	OR912992	OR863910	
*P. neglecta*	TAP1100^T^	*Quercus myrsinaefolia*	Japan	AB482220	LC311600	LC311599	Watanabe et al. (2018)
*P. neolitseae*	NTUCC 17–011^T^	*Neolitsea villosa*	China	MH809383	MH809391	MH809387	Ariyawansa and Hyde (2018)
*P. neolitseae*	CFCC 54590	*Lithocarpus amygdalifolius*	China	OK339744	OK358505	OK358520	Jiang et al. (2022a)
*P. ningboensis*	CFCC 72585^T^	*Pinus elliottii*	China	PV259822	PV275143	PV275217	Wang et al. (2025)
*P. ningboensis*	CFCC 72586	*Pinus elliottii*	China	PV259823	PV275144	PV275218	
*P. novae–hollandiae*	CBS 130973^T^	*Banksia grandis*	Australia	KM199337	KM199511	KM199425	Maharachchikumbura et al. (2014)
*P. oryzae*	CBS 111522	*Telopea* sp.	USA	KM199294	KM199493	KM199394	Maharachchikumbura et al. (2014)
*P. oryzae*	CBS 171.26	NA	Italy	KM199304	KM199494	KM199397	
*P. oryzae*	CBS 353.69^T^	*Oryza sativa*	Denmark	KM199299	KM199496	KM199398	
*P. pallidotheae*	MAFF 240993^T^	*Pieris japonica*	Japan	AB482220	LC311585	LC311584	Watanabe et al. (2010)
*P. pandanicola*	MFLUCC 16–0255^T^	*Pandanus* sp.	Thailand	MH388361	MH388396	MH412723	Tibpromma et al. (2018)
*P. papuana*	CBS 331.96^T^	Coastal soil	Papua New Guinea	KM199321	KM199491	KM199413	Maharachchikumbura et al. (2014)
*P. papuana*	CBS 887.96	*Cocos nucifera*	Papua New Guinea	KM199318	KM199492	KM199415	
*P. parva*	CBS 265.37	*Delonix regia*	NA	KM199312	KM199508	KM199404	Maharachchikumbura et al. (2014)
*P. parva*	CBS 278.35^T^	*Leucothoe fontanesiana*	NA	KM199313	KM199509	KM199405	
*P. photinicola*	GZCC 16–0028^T^	*Photinia serrulata*	China	KY092404	KY047662	KY047663	Chen et al. (2017)
*P. phyllostachydis*	ZHKUCC 23–0873^T^	NA	China	OR343210	OR367675	OR367676	Zhao et al. (2024)
*P. pini*	MEAN 1092^T^	*Pinus pinea*	Portugal	MT374680	MT374693	MT374705	Silva et al. (2020)
*P. pinicola*	KUMCC 19–0183^T^	*Pinus armandii*	China	MN412636	MN417509	MN417507	Tibpromma et al. (2019)
*P. piraubensis*	COAD 2165^T^	*Psidium guajava*	Brazil	MH627381	MH643774	MH643773	Jayawardena et al. (2022)
*P. portugallica*	CBS 393.48^T^	NA	Portugal	KM199335	KM199510	KM199422	Maharachchikumbura et al. (2014)
*P. portugallica*	CBS 684.85	*Camellia japonica*	New Zealand	MH554065	MH554501	MH554741	Liu et al. (2019)
*P. pruni*	CGMCC 3.23507^T^	*Prunus cerasoides*	China	OR248001	OR361476	OR381076	Razaghi et al. (2024)
*P. pruni*	LC15860	*Prunus cerasoides*	China	OR248002	OR361477	OR381077	
*P. rhaphiolepis*	SAUCC367701^T^	*Rhaphiolepis indica*	China	OR733502	OR912994	OR863906	Yin et al. (2024)
*P. rhaphiolepis*	SAUCC367702	*Rhaphiolepis indica*	China	OR733501	OR912995	OR863907	
*P. rhizophorae*	MFLUCC 17–0416^T^	*Rhizophora mucronata*	Thailand	MK764283	MK764327	MK764349	Norphanphoun et al. (2019)
*P. rhizophorae*	MFLUCC 17–0417	*Rhizophora mucronata*	Thailand	MK764284	MK764328	MK764350	
*P. rhododendri*	IFRDCC 2399^T^	*Rhododendron sinogrande*	China	KC537804	KC537811	KC537818	Norphanphoun et al. (2019)
*P. rhodomyrtus*	CFCC 54733	*Quercus aliena*	China	OM746310	OM840082	OM839983	Jiang et al. (2022b)
*P. rhodomyrtus*	CFCC 55052	*Cyclobalanopsis augustinii*	China	OM746311	OM840083	OM839984	
*P. rosarioides*	CGMCC 3.23549^T^	*Rhododendron decorum*	China	OP082430	OP185513	OP185520	Gu et al. (2022)
*P. rosea*	MFLUCC 12–0258^T^	*Pinus* sp.	China	JX399005	JX399069	JX399036	Maharachchikumbura et al. (2012)
*P. rubrae*	CGMCC 3.23499^T^	*Quercus rubra*	China	OR247997	OR361472	OR381072	Razaghi et al. (2024)
*P. rubrae*	LC8233	*Plagiogyria glauca*	China	OR248000	OR361475	OR381075	
*P. sabal*	ZHKUCC 22–0027	*Sabal mexicana*	China	ON180765	ON221523	ON221551	Xiong et al. (2022)
*P. sabal*	ZHKUCC 22–0029	*Sabal mexicana*	China	ON180767	ON221525	ON221553	
*P. schisandrae*	CFCC 59550^T^	*Schisandra sphenanthera*	China	OR775411	OR766002	OR766014	Yuan et al. (2024)
*P. schisandrae*	CFCC 59551	*Schisandra sphenanthera*	China	OR775412	OR766003	OR766015	
*P. scoparia*	CBS 176.25^T^	*Chamaecyparis* sp.	China	KM199330	KM199478	KM199393	Maharachchikumbura et al. (2012)
*P. sequoiae*	MFLUCC 13–0399^T^	*Sequoia sempervirens*	Italy	KX572339	–	–	Maharachchikumbura et al. (2012)
*P. shaanxiensis*	CFCC 54958^T^	*Quercus variabilis*	China	ON007026	ON005043	ON005054	Jiang et al. (2022a)
*P. shaanxiensis*	CFCC 57356	*Quercus variabilis*	China	ON007027	ON005044	ON005055	
*P. shanweiensis*	CFCC 72591^T^	*Pinus massoniana*	China	PV259824	PV275145	PV275219	Wang et al. (2025)
*P. shanweiensis*	CFCC 72592	*Pinus massoniana*	China	PV259825	PV275146	PV275220	
*P. shore*a	MFLUCC 12–0314^T^	*Shorea obtusa*	Thailand	KJ503811	KJ503817	KJ503814	Song et al. (2014)
*P. sichuanensis*	SC3A21^T^	*Camellia sinensis*	China	KX146689	KX146748	KX146807	Wang et al. (2019)
*P. silvicola*	CFCC 55296^T^	*Cyclobalanopsis kerrii*	China	ON007032	ON005049	ON005060	Jiang et al. (2022a)
*P. silvicola*	CFCC 54915	*Cyclobalanopsis kerrii*	China	ON007033	ON005050	ON005061	
*P. silvicola*	CFCC 57363	*Cyclobalanopsis kerrii*	China	ON007034	ON005051	ON005062	
*P. smilacicola*	MFLUCC 22–0125^T^	*Smilax* sp.	Thailand	OP497991	OP753376	OP762673	Sun et al. (2023)
*P. solicola*	SAUCC003804^T^	Soil	China	OQ692020	OQ718737	OQ718795	Li et al. (2024)
*P. solicola*	SAUCC003806	Soil	China	OQ692021	OQ718738	OQ718796	
*P. solicola*	SAUCC003807	Soil	China	OQ692022	OQ718739	OQ718797	
*P. sonneratiae*	CFCC 57392	*Sonneratia apetala*	China	ON114182	ON086810	ON086814	Jiang et al. (2022c)
*P. sonneratiae*	CFCC 57394^T^	*Sonneratia apetala*	China	ON114184	ON086812	ON086816	
*P. sonneratiae*	CFCC 57395	*Sonneratia apetala*	China	ON114185	ON086813	ON086817	
*P. spathulata*	CBS 356.86^T^	*Gevuina avellana*	Chile	KM199338	KM199513	KM199423	Maharachchikumbura et al. (2014)
*P. spathuliappendiculata*	CBS 144035^T^	*Phoenix canariensis*	Australia	MH554172	MH554607	MH554845	Liu et al. (2019)
*P. suae*	CGMCC 3.23546^T^	*Rhododendron delavayi*	China	OP082428	OP185514	OP185521	Gu et al. (2022)
*P. taxicola*	CFCC59976^T^	*Taxus chinensis*	China	OQ626673	OQ714338	OQ714333	Wang et al. (2024)
*P. taxicola*	CFCC59978	*Taxus chinensis*	China	OQ771893	OQ779480	OQ779485	
*P. telopeae*	CBS 114137	*Protea* sp.	Australia	KM199301	KM199559	KM199469	Maharachchikumbura et al. (2014)
*P. telopeae*	CBS 114161^T^	*Telopea* sp.	Australia	KM199296	KM199500	KM199403	
*P. telopeae*	CBS 113606	*Telopea* sp.	Australia	KM199295	KM199498	KM199402	
*P. terricola*	CBS 141.69^T^	Soil	Pacific Islands	MH554004	MH554438	MH554680	Liu et al. (2019)
*P. thailandica*	MFLUCC 17–1616^T^	*Rhizophora apiculata*	Thailand	MK764286	MK764330	MK764352	Maharachchikumbura et al. (2014)
*P. thailandica*	MFLUCC 17–1617	*Rhizophora apiculata*	Thailand	MK764285	MK764329	MK764351	
*P. thunbergii*	CFCC 72589^T^	*Pinus thunbergii*	China	PV259838	PV275159	PV275233	Wang et al. (2025)
*P. thunbergii*	CFCC 72590	*Pinus thunbergii*	China	PV259839	PV275160	PV275234	
*P. trachycarpicola*	OP068^T^	*Trachycarpus fortunei*	China	JQ845947	JQ845946	JQ845945	Zhang et al. (2012b)
*P. trachycarpicola*	OP143	*Podocarpus macrophyllus*	China	KC537809	KC537816	KC537823	
*P. trachycarpicola*	MFLUCC 12-0267	Unidentified tree	China	JX399001	JX399065	JX399032	Maharachchikumbura et al. (2012)
*P. tumida*	CFCC 55158^T^	*Rosa chinensis*	China	OK560610	OL814524	OM158174	Peng et al. (2022)
*P. tumida*	CFCC 55159	*Rosa chinensis*	China	OK560613	OL814527	OM158177	
*P. unicolor*	MFLUCC 12–0276^T^	*Rhododendron* sp.	China	JX398999	–	JX399030	Maharachchikumbura et al. (2012)
*P. verruculosa*	MFLUCC 12–0274^T^	*Rhododendron* sp.	China	JX398996	JX399061	–	Maharachchikumbura et al. (2012)
*P. wenzhouensis*	CFCC 72587^T^	*Pinus massoniana*	China	PV259840	PV275161	PV275235	Wang et al. (2025)
*P. wenzhouensis*	CFCC 72588	*Pinus massoniana*	China	PV259845	PV275166	PV275240	
*P. wulichongensis*	CGMCC 3.23469^T^	Poaceae	China	OR247978	OR361453	OR381053	Razaghi et al. (2024)
*P. wulichongensis*	LC15846	Poaceae	China	OR247979	OR361454	OR381054	
*P. yanglingensis*	LC 4553^T^	*Camellia sinensis*	China	KX895012	KX895231	KX895345	Liu et al. (2017)
*P. yunnanensis*	HMAS 96359^T^	*Podocarpus macrophyllus*	China	AY373375	–	–	Wei et al. (2013)
*Nonappendiculata quercina*	CBS 116061^T^	*Quercus suber*	Italy	MH553982	MH554400	MH554641	Liu et al. (2019)
*N. quercina*	CBS 270.82	*Quercus pubescens*	Italy	MH554025	MH554459	MH554701	

The ex-type strains are indicated using “^T^” after strain numbers; “–” stands for no sequence data in GenBank.

### Phylogenetic analyses

According to recent publications (Hsu et al. 2024; Li et al. 2024; Luo et al. 2024; Zhao et al. 2024; Cao et al. 2025; Jiang et al. 2025; Wang et al. 2025), sequences of *Pestalotiopsis* species obtained from GenBank were selected for phylogenetic analyses together with the 21 strains in this study (Table 2). *Nonappendiculata quercina* (CBS 116061 and CBS 270.82) was used as the outgroup. All sequences were aligned using MAFFT v.7.526 (Katoh et al. 2019, https://mafft.cbrc.jp/alignment/server/). A combined multilocus sequence dataset comprising ITS, *tef*1-α, and *tub*2 was assembled and manually optimized to improve alignment and sequence accuracy using PhyloSuite v.1.2.2 (Zhang et al. 2020). Phylogenetic trees were constructed based on the concatenated dataset using maximum likelihood (ML) and Bayesian inference (BI) in PhyloSuite v.1.2.2 (Zhang et al. 2020). The optimal partition model (edge-linked) was selected using the ModelFinder function (Kalyaanamoorthy et al. 2017), with the BIC criterion applied for IQ-TREE construction and the AICc criterion implemented for MrBayes analysis. Maximum likelihood phylogenies were constructed using IQ-TREE (Nguyen et al. 2015) under an edge-linked partition model, with nodal support assessed through 10,000 ultrafast bootstrap replicates (Minh et al. 2013). The final phylogenetic tree was selected from suboptimal trees of each run through likelihood score comparison, with the following models applied to respective gene partitions: HKY+F+R5 for ITS, TIM2e+R4 for *tef*1-α, and TPM2u+F+I+G4 for *tub*2. Bayesian inference phylogenies were constructed using MrBayes v.3.2.6 (Ronquist et al. 2012) under a partition model configuration, with two parallel runs and 2,000,000 generations, while discarding the initial 25% of sampled data as burn-in. The best-fit models were HKY+F+I+G4 for ITS, GTR+F+G4 for *tef*1-α, and GTR+F+I+G4 for *tub*2. Phylogenetic trees were visualized using FigTree v.1.4.4 (http://tree.bio.ed.ac.uk/software/figtree), with subsequent editing and layout finalized in Adobe Illustrator CS v.5.

## Results

### Molecular phylogeny

**Figure 1.**
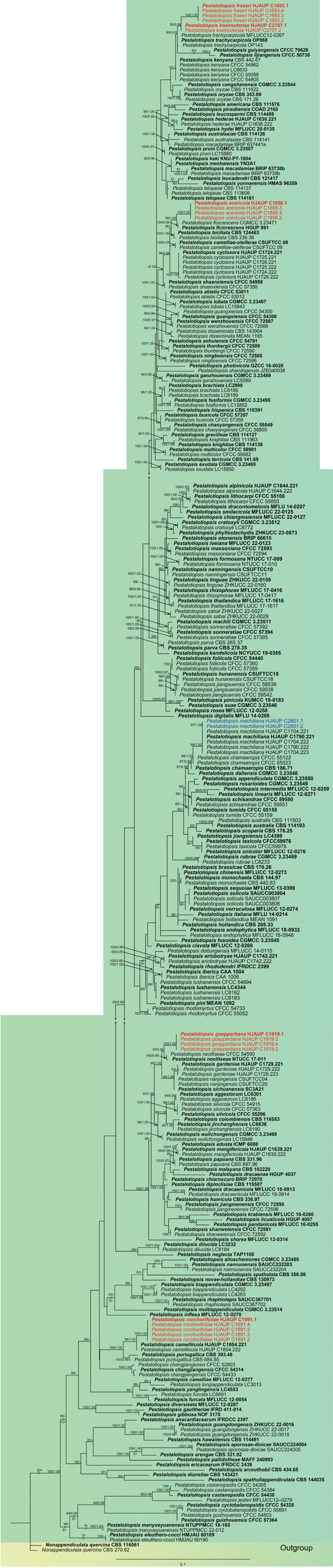
A maximum-likelihood phylogenetic tree of *Pestalotiopsis* constructed based on concatenated sequence data of ITS, *tef*1-α, and *tub*2. Bootstrap support values for ML greater than 80% and Bayesian posterior probabilities greater than 0.95 are shown near the nodes. The tree is rooted with *Nonappendiculata quercina* (CBS 116061 and CBS 270.82). New strains identified in this study are shown in blue; new species are shown in red. Some branches were shortened according to the indicated multipliers, and these are indicated by the symbol (**//**).

Based on the concatenated sequences of the three barcode genes (ITS, *tef*1-α, and *tub*2), the phylogenetic tree (Fig. 1) was constructed using maximum likelihood (ML) and Bayesian inference (BI) to analyze the phylogenetic relationships of the 21 strains within the genus *Pestalotiopsis*. The phylogenetic analyses included 301 strains (Table 1), comprising 159 accepted *Pestalotiopsis* species and 21 strains from this study, with *Nonappendiculata quercina* (CBS 116061 and CBS 270.82) serving as the outgroup. The combined sequence consisted of 1472 nucleotide positions (ITS: 1–556, *tef*1-α: 557–1013, *tub*2: 1014–1472), comprising 901 distinct patterns, 662 parsimony-informative sites, 163 singleton sites, and 647 constant sites. Phylogenetic trees were constructed based on the concatenated sequences using ML and BI methods with essentially identical topological architectures. The optimal ML phylogenetic tree (lnL = −19450.797) is shown in Fig. 1, with node support values annotated adjacent to each branch. At each node, the first and second numerical descriptors correspond to ultrafast bootstrap support values from ML and posterior probabilities obtained from BI, respectively.

### Taxonomy

#### Pestalotiopsis acericola

Taxon classificationFungiAmphisphaerialesPestalotiopsidaceae

M.G. Liao & Jian Ma sp. nov.

7D2EAFC3-3BC4-575C-A1F2-7CE493E1F903

Index Fungorum: IF904707

Fig. 2

##### Type.

China • Zhejiang Province, Hangzhou City, West Lake Scenic Area, on diseased leaves of *Acer buergerianum*, 22 October 2023, X.X. Luo (HJAUP M1856, ***holotype***), ex-type living culture, HJAUP C1856.1 = HJAUP C1856.2 = HJAUP C1856.3 = HJAUP C1856.4.

##### Etymology.

Named after the genus, *Acer*, from which the fungus was isolated.

##### Description.

Regular leaf spots, grey white in center with blurred margin. **Asexual morph on PDA**: Conidiomata acervular, globose to clavate, 350–660 μm diam., superficial, solitary or aggregated in clusters, black. Conidiophores indistinct and reduced to conidiogenous cells. Conidiogenous cells hyaline, smooth, cylindrical to ampulliform. Conidia fusiform, straight or slightly curved, 16.1–24.5 × 5.2–6.9 μm (x̄ = 20.9 × 5.9 μm, n = 50), 4-septate, slightly constricted at the septa; basal cell conical, 3.1–5.3 μm (x̄ = 4.1 μm), hyaline or sometimes pale brown, smooth, thin-walled, with a single filiform appendage, unbranched, 4.6–10.7 μm (x̄ = 7.0 μm) long; three median cells doliiform to cylindrical, smooth, thick-walled, 10.4–14.6 μm (x̄ = 12.8 μm), concolorous, pale brown to brown, somewhat constricted at the septa, second cell from the base 3.6–5.0 µm (x̄ = 4.4 μm) long, third cell 3.0–5.0 µm (x̄ = 4.1 μm) long, fourth cell 3.6–5.8 µm (x̄ = 4.6 μm) long); apical cell conical to acute, hyaline, smooth, thin-walled, 2.6–4.6 µm (x̄ = 3.9 μm) long, with 2–3 filiform appendages, arising from the apex of the apical cell each at a different point, unbranched, 12.3–27.5 µm (x̄ = 17.7 μm) long. **Sexual morph**: not observed.

**Figure 2.**
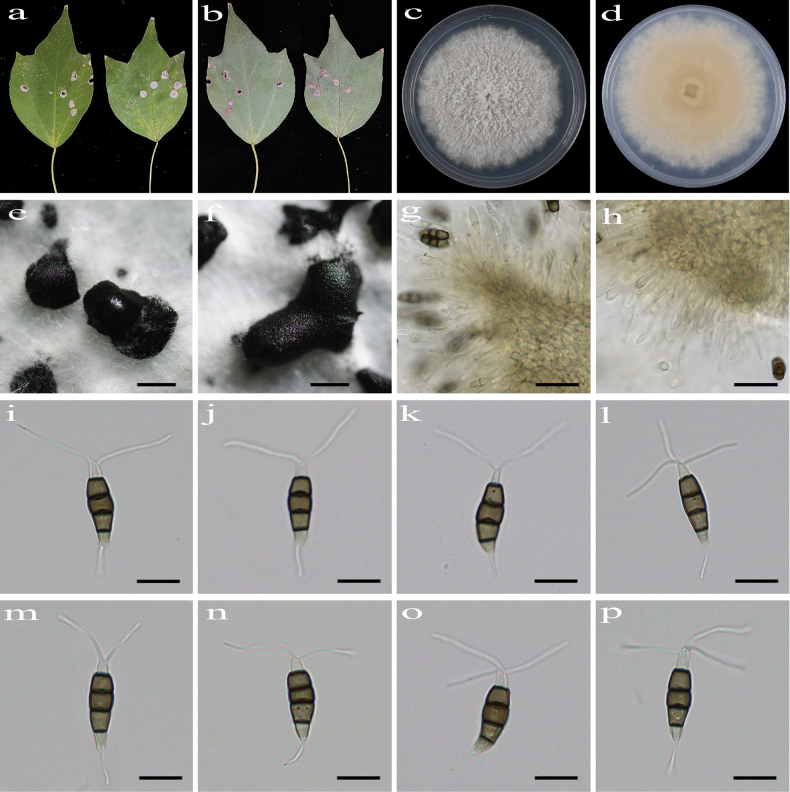
*Pestalotiopsis acericola* (HJAUP C1856.1, ex-type). **a, b**. Leaf of host plant (front and reverse); **c, d**. Culture on PDA (front and reverse); **e, f**. Conidiomata; **g, h**. Conidiogenous cells and conidia; **i–p**. Conidia. Scale bars: 400 µm (**e, f**); 20 µm (**g, h**); 10 µm (**i–p**).

##### Culture characteristics.

Colonies on PDA grow fast, filamentous, reaching 76–82 mm diam. after 5–7 days at 25 °C in light and dark conditions (8 h/16 h), white, with flocculent mycelium and entire edge, forming black, brown conidiomata, and reverse pale orange in center.

##### Note.

Phylogenetic analyses revealed that the four strains (HJAUP C1856.1^T^, HJAUP C1856.2, HJAUP C1856.3, and HJAUP C1856.4) of *P. acericola* form a distinct clade sister to *P. ficicrescens* (HGUP 861^T^ and CGMCC 3.23471) with 98% ML/1.00 BI bootstrap support (Fig. 1). Based on a BLASTn search of *P. acericola* (HJAUP C1856.1^T^) and *P. ficicrescens* (HGUP 861^T^) in GenBank, comparisons of nucleotides showed 2 bp (537/539, including two gaps), 1 bp (291/292, no gaps), and 4 bp (451/455, including one gap) nucleotide differences in the ITS, *tef*1-α, and *tub*2 regions, respectively. Moreover, *P. acericola* differs from *P. ficicrescens* Qi Yang & Yong Wang bis (Hyde et al. 2023) in its smaller conidiomata (350–660 μm diam. vs. 400–1200 μm diam.) and wider conidia (5.2–6.9 μm vs. 3–5.5 μm) with a longer fourth cell (3.6–5.8 µm vs. 3–5 µm) from the base, a longer basal appendage (4.6–10.7 μm vs. 3.5–7 µm), and longer apical appendages (12.3–27.5 μm vs. 10.5–18 µm). Furthermore, *P. acericola* can be distinguished from *P. ficicrescens* by its different host (*Acer buergerianum* vs. *Ficus tikoua*).

#### Pestalotiopsis corchorifolii

Taxon classificationFungiAmphisphaerialesPestalotiopsidaceae

M.G. Liao & Jian Ma sp. nov.

AA172086-4AAB-5E13-A437-236403FC8465

861554

Fig. 3

##### Type.

China • Zhejiang Province, Wenzhou City, Wenzhou Botanical Garden, on diseased leaves of *Rubus corchorifolius*, 24 October 2023, X.X. Luo (HJAUP M1891, ***holotype***), ex-type living culture HJAUP C1891.1 = HJAUP C1891.2 = HJAUP C1891.3 = HJAUP C1891.4 = HJAUP M1891.5.

##### Etymology.

Named after the host species, *Rubus corchorifolius*, from which the fungus was isolated.

##### Description.

Regular leaf spots, grey white in center with brown at margin. **Asexual morph on PDA**: Conidiomata acervular, globose to clavate, 849–1375 μm diam., solitary or aggregated in clusters, black. Conidiophores indistinct and reduced to conidiogenous cells. Conidiogenous cells hyaline, smooth, cylindrical to ampulliform. Conidia fusiform, straight or slightly curved, 14.6–21.6 × 4.4–5.9 μm (x̄ = 17.4 × 5.3 μm, n = 50), 4-septate, slightly constricted at the septa; basal cell conical, 2.2–3.9 μm (x̄ = 2.6 μm), hyaline, smooth, thin-walled, with a single filiform appendage, unbranched, 0.9–3.1 μm (x̄ = 2.1 μm) long; three median cells doliiform to cylindrical, smooth, thick-walled, 10.5–14.0 μm (x̄ = 12.1 μm), concolorous, brown, somewhat constricted at the septa, second cell from the base 3.4–5.6 µm (x̄ = 4.1 μm) long, third cell 3.3–4.6 µm (x̄ = 4.1 μm) long, fourth cell 3.4–4.7 µm (x̄ = 4.0 μm) long); apical cell conical to acute, hyaline, smooth, thin-walled, 1.9–3.7 µm (x̄ = 2.7 μm) long, with 2–3 filiform appendages, arising from the apex of the apical cell each at a different point, unbranched, 6.7–17.4 µm (x̄ = 11.5 μm) long. **Sexual morph**: not observed.

**Figure 3.**
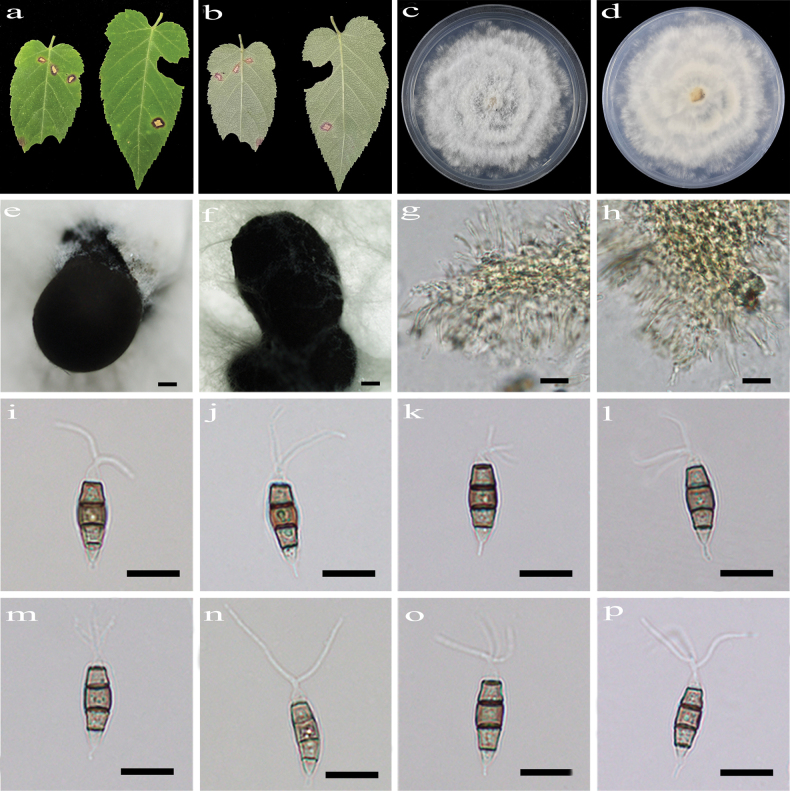
*Pestalotiopsis corchorifolii* (HJAUP C1891.1, ex-type). **a, b**. Leaf of host plant (front and reverse); **c, d**. Culture on PDA (front and reverse); **e, f**. Conidiomata; **g, h**. Conidiogenous cells and conidia; **i–p**. Conidia. Scale bars: 200 µm (**e, f**); 10 µm (**g–p**).

##### Culture characteristics.

Colonies on PDA grow fast, filamentous, reaching 85–90 mm diam. after 5–7 days at 25 °C in light and dark conditions (8 h/16 h), white, with flocculent mycelium and entire edge, forming black conidiomata, and reverse white.

##### Note.

Phylogenetic analyses revealed that the five strains (HJAUP C1891.1^T^, HJAUP C1891.2, HJAUP C1891.3, HJAUP C1891.4, and HJAUP M1891.5) of *P. corchorifolii* form a distinct clade sister to *P. camelliicola* (HJAUP C1804.221^T^ and HJAUP C1804.222) with 98% ML/1.00 BI bootstrap support (Fig. 1). Based on a BLASTn search of *P. corchorifolii* (HJAUP C1891.1^T^) and *P. camelliicola* (HJAUP C1804.221^T^) in GenBank, comparisons of nucleotides showed 1 bp (630/631, including one gap), 5 bp (296/301, including three gaps), and 5 bp (476/481, including one gap) nucleotide differences in the ITS, *tef*1-α, and *tub*2 regions, respectively. Moreover, *P. corchorifolii* can be distinguished from *P. camelliicola* X.X. Luo & Jian Ma (Luo et al. 2024) by its larger conidiomata (849–1375 μm diam. vs. 470–1320 μm diam.) and smooth, narrower conidia (4.4–5.9 μm vs. 5.7–7.6 μm), and further differs in that the conidia of *P. camelliicola* have 2–4 tubular apical appendages arising from an apical crest or irregularly branched along their length, resulting in 2–3 branches. Furthermore, *P. corchorifolii* can be distinguished from *P. camelliicola* by its different host (*Rubus corchorifolius* vs. *Camellia japonica*).

#### Pestalotiopsis fraseri

Taxon classificationFungiAmphisphaerialesPestalotiopsidaceae

M.G. Liao & Jian Ma sp. nov.

A3BC8BEE-4D82-5832-AE7F-6DD8C75996A6

861555

Fig. 4

##### Type.

China • Jiangxi Province, Nanchang City, Jiangxi Agricultural University, on diseased leaves of *Photinia × fraseri* Dress, 26 October 2023, X.X. Luo (HJAUP M1693, ***holotype***), ex-type living culture HJAUP C1693.1 = HJAUP C1693.2 = HJAUP C1693.3 = HJAUP C1693.4.

##### Etymology.

Named after the host species, *Photinia × fraseri*, from which the fungus was isolated.

##### Description.

Regular leaf spots, pale brown in center with brown at margin. **Asexual morph on PDA**: Conidiomata acervular, globose to clavate, 426–786 μm diam., solitary, black. Conidiophores indistinct and reduced to conidiogenous cells. Conidiogenous cells hyaline, smooth, cylindrical to ampulliform. Conidia fusiform, straight or slightly curved, 19.3–26.4 × 5.2–6.3 μm (x̄ = 23.0 × 5.7 μm, n = 50), 4-septate, slightly constricted at the septa; basal cell conical, 3.4–5.3 μm (x̄ = 4.4 μm), hyaline, smooth, thin-walled, with a single filiform appendage, unbranched, 4.6–10.5 μm (x̄ = 7.9 μm) long; three median cells doliiform to cylindrical, smooth, thick-walled, 12.2–16.2 μm (x̄ = 14.4 μm), concolorous, brown, somewhat constricted at the septa, second cell from the base 3.9–6.1 µm (x̄ = 4.8 μm) long, third cell 4.0–5.4 µm (x̄ = 4.7 μm) long, fourth cell 4.0–5.2 µm (x̄ = 4.7 μm) long); apical cell conical to acute, hyaline, smooth, thin-walled, 3.7–5.0 µm (x̄ = 4.2 μm) long, with 2–3 filiform appendages, arising from the apex of the apical cell each at a different point, unbranched, 12.3–20.8 µm (x̄ = 16.3 μm) long. **Sexual morph**: not observed.

**Figure 4.**
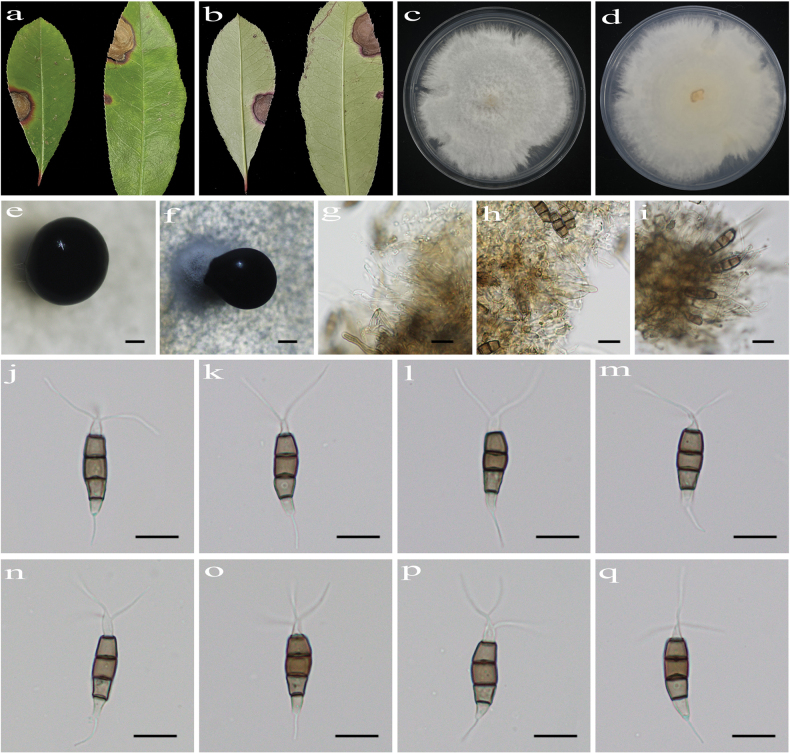
*Pestalotiopsis fraseri* (HJAUP C1693.1, ex-type). **a, b**. Leaf of host plant (front and reverse); **c, d**. Culture on PDA (front and reverse); **e, f**. Conidiomata; **g–i**. Conidiogenous cells and conidia; **j–q**. Conidia. Scale bars: 200 µm (**e, f**); 10 µm (**g–q**).

##### Culture characteristics.

Colonies on PDA grow fast, filamentous, reaching 85–90 mm diam. after 5–7 days at 25 °C in light and dark conditions (8 h/16 h), white, with flocculent mycelium and entire edge, forming black conidiomata, and reverse white.

##### Note.

Phylogenetic analyses revealed that the four strains (HJAUP C1693.1^T^, HJAUP C1693.2, HJAUP C1693.3, and HJAUP C1693.4) of *P. fraseri* form a distinct clade sister to *P. koelreuteriae* (HJAUP C2757.1^T^ and HJAUP C2757.2) with 100% ML/1.00 BI bootstrap support (Fig. 1). Based on a BLASTn search of *P. fraseri* (HJAUP C1693.1^T^) and *P. koelreuteriae* (HJAUP C2757.1^T^) in GenBank, comparisons of nucleotides showed 13 bp (619/632, including three gaps), 1 bp (296/297, no gaps), and 3 bp (479/482, including one gap) nucleotide differences in the ITS, *tef*1-α, and *tub*2 regions, respectively. Based on a BLASTn search of *P. fraseri* (HJAUP C1693.1^T^) and *P. trachycarpicola* (OP068^T^) in GenBank, comparisons of nucleotides showed 10 bp (530/540, including one gap) and 1 bp (294/295, no gaps) in the ITS and *tef*1-α regions, respectively. Moreover, *P. fraseri* differs from *P. koelreuteriae* (this study) in its conidia with longer basal appendages (4.6–10.5 μm vs. 3.4–6.7 μm) and longer apical appendages (12.3–20.8 µm vs. 8.1–18.5 µm), and from *P. trachycarpicola* Yan M. Zhang & K.D. Hyde (Zhang et al. 2012b) in its longer basal appendages (4.6–10.5 μm vs. 2.7–5.5 μm). Furthermore, *P. fraseri* can be distinguished from *P. koelreuteriae* and *P. trachycarpicola* by its different host (*Photinia × fraseri* vs. *Koelreuteria paniculata* vs. *Trachycarpus fortunei*).

#### Pestalotiopsis goeppertiae

Taxon classificationFungiAmphisphaerialesPestalotiopsidaceae

M.G. Liao & Jian Ma sp. nov.

CF296B09-00B2-5B7A-BE1A-CE5610216FC1

861556

Fig. 5

##### Type.

China • Fujian Province, Fuzhou City, Fuzhou Botanical Garden, on diseased leaves of *Goeppertia makoyana*, 24 October 2023, X.X. Luo (HJAUP M1919, ***holotype***), ex-type living culture HJAUP C1919.1 = HJAUP C1919.2 = HJAUP C1919.3 = HJAUP C1919.4.

##### Etymology.

Named after the genus, *Goeppertia*, from which the fungus was isolated.

##### Description.

Regular leaf spots, grey white in center with brown at margin. Asexual morph on PDA: Conidiomata acervular, globose to clavate, 550–1520 μm diam., superficial, solitary or aggregated in clusters, dark brown. Conidiophores indistinct and reduced to conidiogenous cells. Conidiogenous cells hyaline, smooth, cylindrical to ampulliform. Conidia fusiform, straight or slightly curved, 17.0–23.6 × 5.0–5.9 μm (x̄ = 19.5 × 5.5 μm, n = 50), 4-septate, slightly constricted at the septa; basal cell conical, 2.8–4.5 μm (x̄ = 3.5 μm), hyaline or sometimes pale brown, smooth, thin-walled, with a single filiform appendage, unbranched, 2.3–6.0 μm (x̄ = 3.5 μm) long; three median cells doliiform to cylindrical, smooth, thick-walled, 11.0–14.5 μm (x̄ = 12.4 μm), concolorous, pale brown to brown, somewhat constricted at the septa, second cell from the base 3.4–5.1 µm (x̄ = 4.2 μm) long, third cell 3.7–4.6 µm (x̄ = 4.2 μm) long, fourth cell 3.7–4.9 µm (x̄ = 4.2 μm) long); apical cell conical to acute, hyaline, smooth, thin-walled, 3.2–4.1 µm (x̄ = 3.7 μm) long, with 2–3 filiform appendages, arising from the apex of the apical cell each at a different point, unbranched, 12.6–21.3 µm (x̄ = 15.4 μm) long. **Sexual morph**: not observed.

**Figure 5.**
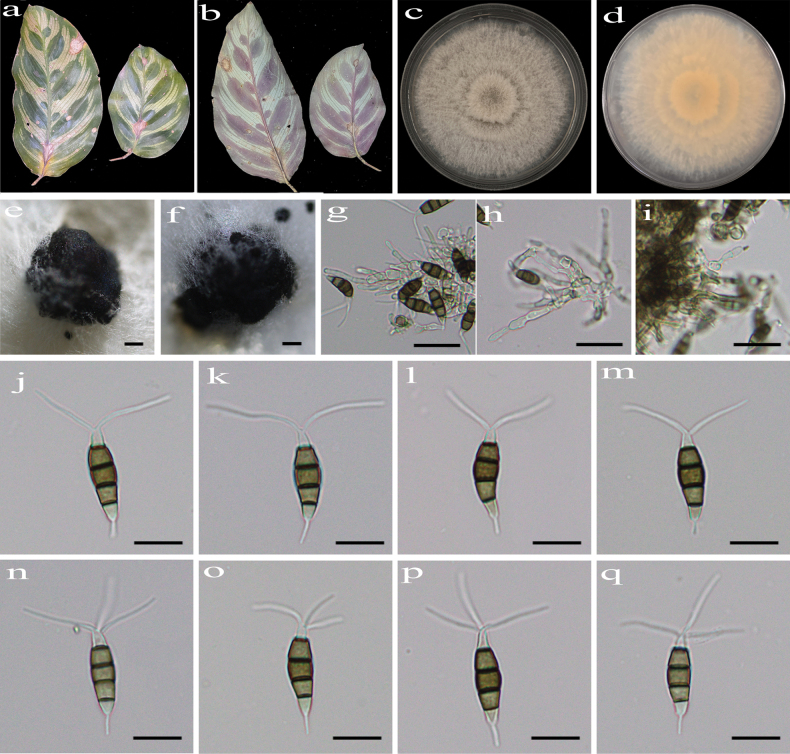
*Pestalotiopsis goeppertiae* (HJAUP C1919.1, ex-type). **a, b**. Leaf of host plant (front and reverse); **c, d**. Culture on PDA (front and reverse); **e, f**. Conidiomata; **g–i**. Conidiogenous cells and conidia; **j–q**. Conidia. Scale bars: 200 µm (**e, f**); 20 µm (**g–i**); 10 µm (**j–q**).

##### Culture characteristics.

Colonies on PDA grow fast, filamentous, reaching 85–90 mm diam. after 5–7 days at 25 °C in light and dark conditions (8 h/16 h), white, with flocculent mycelium and entire edge, forming black, brown conidiomata, and reverse pale orange.

##### Note.

Phylogenetic analyses revealed that the four strains (HJAUP C1919.1^T^, HJAUP C1919.2, HJAUP C1919.3, and HJAUP C1919.4) of *P. goeppertiae* form a distinct clade sister to *P. neolitseae* (NTUCC 17–011^T^ and CFCC 54590) with 97% ML/0.96 BI bootstrap support (Fig. 1). Based on a BLASTn search of *P. goeppertiae* (HJAUP C1919.1^T^) and *P. neolitseae* (NTUCC 17–011^T^) in GenBank, comparisons of nucleotides showed 9 bp (248/257, including four gaps) and 1 bp (399/400, no gaps) nucleotide differences in the *tef*1-α and *tub*2 regions, respectively. Moreover, *P. goeppertiae* differs from *P. neolitseae* H.A. Ariy. & K.D. Hyde (Ariyawansa and Hyde 2018) in its conidia with a shorter fourth cell (3.7–4.9 µm vs. 4–5(–6) µm) from the base and longer apical appendages (12.6–21.3 µm vs. (7–)10–15(–17) μm). Furthermore, *P. goeppertiae* can be distinguished from *P. neolitseae* by its different host (*Goeppertia makoyana* vs. *Neolitsea villosa*).

#### Pestalotiopsis koelreuteriae

Taxon classificationFungiAmphisphaerialesPestalotiopsidaceae

M.G. Liao & Jian Ma sp. nov.

DF7962FF-410E-5E7A-9B5D-2F6BF761096F

861557

Fig. 6

##### Type.

China • Hunan Province, Zhangjiajie City, Zhangjiajie National Forest Park, on diseased leaves of *Koelreuteria paniculata*, 18 October 2024, M.G. Liao (HJAUP M2757, ***holotype***), ex-type living culture HJAUP C2757.1 = HJAUP C2757.2.

##### Etymology.

Named after the genus, *Koelreuteria*, from which the fungus was isolated.

##### Description.

Irregular leaf spots, pale brown in center with brown at margin. Asexual morph on PDA: Conidiomata acervular, globose to clavate, 610–750 μm diam., solitary or aggregated in clusters, black. Conidiophores indistinct and reduced to conidiogenous cells. Conidiogenous cells hyaline, smooth, cylindrical to ampulliform. Conidia fusiform, straight or slightly curved, 18.3–26.9 × 4.1–6.0 μm (x̄ = 22.7 × 5.3 μm, n = 50), 4-septate, slightly constricted at the septa; basal cell conical, 2.7–4.6 μm (x̄ = 3.9 μm), hyaline or sometimes pale brown, smooth, thin-walled, with a single filiform appendage, unbranched, 3.4–6.7 μm (x̄ = 4.9 μm) long; three median cells doliiform to cylindrical, smooth, thick-walled, 12.8–17.3 μm (x̄ = 15.2 μm), concolorous, brown, somewhat constricted at the septa, second cell from the base 3.4–6.6 µm (x̄ = 4.8 μm) long, third cell 3.4–6.5 µm (x̄ = 5.3 μm) long, fourth cell 3.8–6.6 µm (x̄ = 5.2 μm) long); apical cell conical to acute, hyaline, smooth, thin-walled, 2.5–5.1 µm (x̄ = 3.5 μm) long, with 1–3 (mostly 2) filiform appendages, arising from the apex of the apical cell each at a different point, unbranched, 8.1–18.5 µm (x̄ = 13.2 μm) long. **Sexual morph**: not observed.

**Figure 6.**
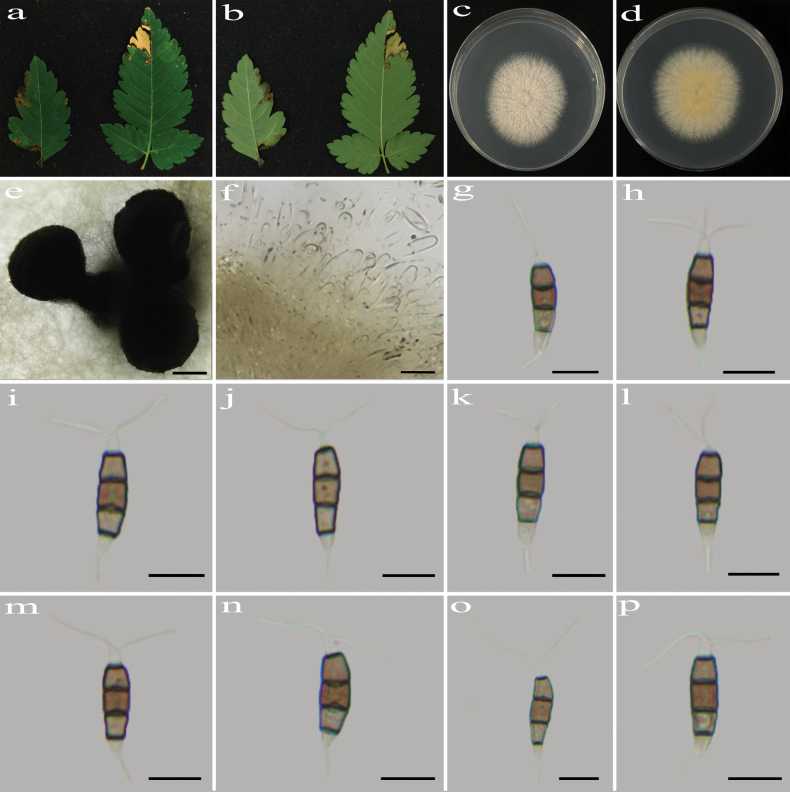
*Pestalotiopsis koelreuteriae* (HJAUP C2757.1, ex-type). **a, b**. Leaf of host plant (front and reverse); **c, d**. Culture on PDA (front and reverse); **e**. Conidiomata; **f**. Conidiogenous cells and conidia; **g–p**. Conidia. Scale bars: 200 µm (**e**); 10 µm (**f–p**).

##### Culture characteristics.

Colonies on PDA grow fast, filamentous, reaching 50–60 mm diam. after 5–7 days at 25 °C in light and dark conditions (8 h/16 h), white, with flocculent mycelium and entire edge, forming black conidiomata, and reverse pale orange.

##### Note.

Phylogenetic analyses revealed that the two strains (HJAUP C2757.1^T^ and HJAUP C2757.2) of *P. koelreuteriae* form a distinct clade sister to *P. fraseri* (HJAUP C1693.1^T^, HJAUP C1693.2, HJAUP C1693.3, and HJAUP C1693.4) with 100% ML/1.00 BI bootstrap support (Fig. 1). The nucleotide divergences and morphological differentiations between *P. koelreuteriae* and *P. fraseri* have been delineated in the preceding section. Comparisons of nucleotides showed 13 bp (2.1%, including three gaps) in the ITS region. Based on the criteria established by Jeewon and Hyde (2016), a divergence of more than 1.5% in the ITS nucleotide sequence can support the recognition of a novel species. Thus, *P. koelreuteriae* was proposed as a new species.

#### Pestalotiopsis machiliana

Taxon classificationFungiAmphisphaerialesPestalotiopsidaceae

X.X. Luo & Jian Ma, Mycokeys 109: 229 (2024)

DC673386-6F04-529D-AE51-5556306EEC4B

Index Fungorum: IF902325

Fig. 7

##### Type.

China • Hubei Province, Wuhan City, Wuhan Botanical Garden, on diseased leaves of *Bambusa multiplex* (Lour.) Raeusch. ex Schult. cv. Fernleaf R. A. Young, 24 October 2024, M.G. Liao (HJAUP M2801, living culture HJAUP C2801.1 = HJAUP C2801.2).

##### Description.

Leaf tip lesions, paler brown spots with brown margin between diseased and healthy tissue. **Asexual morph on PDA**: Conidiomata acervular, globose to clavate, 1000–1350 μm diam., solitary or aggregated in clusters, black. Conidiophores indistinct and reduced to conidiogenous cells. Conidiogenous cells hyaline, smooth, cylindrical to ampulliform. Conidia fusiform, straight or slightly curved, 18.1–25.1 × 4.9–6.8 μm (x̄ = 21.3 × 5.8 μm, n = 50), 4-septate, slightly constricted at the septa; basal cell conical, 2.9–5.3 μm (x̄ = 3.9 μm), hyaline or sometimes pale brown, smooth, thin-walled, with a single filiform appendage, unbranched, 4.4–7.1 μm (x̄ = 5.9 μm) long; three median cells doliiform to cylindrical, smooth, thick-walled, 11.2–17.3 μm (x̄ = 13.9 μm), concolorous, brown, somewhat constricted at the septa, second cell from the base 3.5–5.7 µm (x̄ = 4.6 μm) long, third cell 3.9–5.9 µm (x̄ = 4.7 μm) long, fourth cell 3.8–5.7 µm (x̄ = 4.6 μm) long); apical cell conical to acute, hyaline, smooth, thin-walled, 2.3–4.8 µm (x̄ = 3.5 μm) long, with 2–3 filiform appendages, arising from the apex of the apical cell each at a different point, unbranched, 9.3–20.8 µm (x̄ = 14.9 μm) long. **Sexual morph**: not observed.

**Figure 7.**
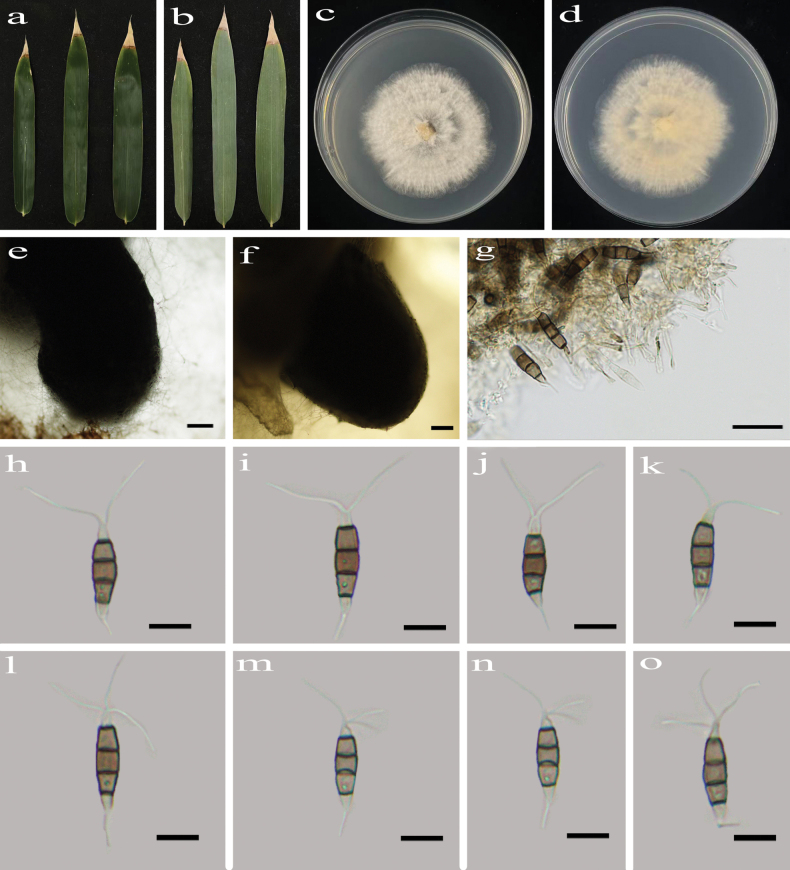
*Pestalotiopsis machiliana* (HJAUP C2801.1). **a, b**. Leaf of host plant (front and reverse); **c, d**. Culture on PDA (front and reverse); **e–f**. Conidiomata; **g**. Conidiogenous cells and conidia; **h–o**. Conidia. Scale bars: 200 µm (**e, f**); 10 µm (**g–o**).

##### Culture characteristics.

Colonies on PDA grow fast, filamentous, reaching 55–70 mm diam. after 5–7 days at 25 °C in light and dark conditions (8 h /16 h), white, with flocculent mycelium and entire edge, forming black conidiomata, and reverse pale orange.

##### Notes.

*Pestalotiopsis machiliana* was introduced by Luo et al. (2024) and isolated from diseased leaves of *Machilus pauhoi* in Jiangxi Province, China. Phylogenetic analyses showed that our new isolates (HJAUP C2801.1 and HJAUP C2801.2) cluster with the group of *P. machiliana* (HJAUP C1704.221, HJAUP C1704.222, HJAUP C1704.223, HJAUP C1790.221^T^, and HJAUP C1790.222) with 99% ML/1.00 BI bootstrap support (Fig. 1). A BLASTn search of GenBank revealed that the sequences of our new isolate (HJAUP C2801.1) and *P. machiliana* (HJAUP C1790.221^T^) share 99% similarity (630/632, including one gap) in ITS, 100% similarity (272/272, no gaps) in *tef*1-α, and 99% similarity (481/482, including one gap) in *tub*2. Morphologically, our isolate aligns well with the original description of *P. machiliana* (Luo et al. 2024). Thus, based on the high morphological similarity and only minor molecular differences, we identified our new isolate as *P. machiliana*.

## Discussion

Morphological characteristics have consistently served as a fundamental criterion for the identification of *Pestalotiopsis* species. However, with the continuous increase in the number of *Pestalotiopsis* species, the criteria used for species identification have become increasingly ambiguous (Huanaluek et al. 2021). Maharachchikumbura et al. (2014) previously suggested that continued reliance on conidial size for species delineation within *Pestalotiopsis* is methodologically unsound. In this study, no significant differences were observed in conidial size between *P. acericola* and *P. chiangmaiensis* Y.R. Sun & Yong Wang bis (16.1–24.5 × 5.2–6.9 µm vs. 16–26 × 4–7 µm; Sun et al. 2023), but the two species were clustered into different branches, and the nucleotide differences between *P. acericola* (HJAUP C1856.1^T^) and *P. chiangmaiensis* (MFLUCC 22–0127^T^) showed 0.8% (560/565, one gap) in ITS, 7.4% (250/270, eight gaps) in *tef*1-α, and 3.5% (440/456, one gap) in *tub*2. Sun et al. (2023) proposed that the individual cell length characteristics of the three median cells should not be considered criteria for interspecific differentiation, as individual cell length measurements duplicate the description of the total length of the three median cells. Therefore, it was suggested that individual cell length measurements be removed from morphological descriptions to achieve simplification. Additionally, based on phylogenetic analyses, *P. exudata* P. Razaghi, F. Liu & L. Cai and *P. multiappendiculata* P. Razaghi, F. Liu & L. Cai were proposed as novel species (Razaghi et al. 2024), but their conidial septation (4–5-septate) is inconsistent with the generally accepted morphological concept of 5-celled conidia for *Pestalotiopsis* species. Although phylogenetic analyses together with morphology currently serve as an effective approach for identifying *Pestalotiopsis* species, ambiguous morphological concepts can render the classification of these taxa questionable.

To date, 457 species of *Pestalotiopsis* have been recorded in Index Fungorum (Index Fungorum 2025), but molecular data are available in GenBank for only 159 species. The absence of molecular data precludes the resolution of interspecific relationships through phylogenetic analyses and results in substantial ambiguity in the taxonomic placement of morphologically congruent species within the genus. Maharachchikumbura et al. (2012) tested 10 gene regions (*ACT*, *tub2*, *CAL*, *GPDH*, *GS*, ITS, LSU, *RPB1*, SSU, and *tef*1-α) to resolve cryptic *Pestalotiopsis* species and ultimately selected ITS, *tef*1-α, and *tub*2 as the most effective molecular markers. However, recent phylogenetic analyses based on these three markers revealed that some species, such as *P. verruculosa* (MFLUCC 12–0274), lacking *tub*2, and *P. chinensis* (MFLUCC 12–0273), *P. sequoiae* (MFLUCC 13–0399), and *P. yunnanensis* (HMAS 96359), lacking *tub*2 and *tef*1-α, showed low support values (Hsu et al. 2024; Li et al. 2024; Yin et al. 2024). The low support values for these taxa may be attributed to the absence of *tef*1-α and/or *tub*2 sequence data, which likely resulted in insufficient phylogenetic information. Thus, completing the ITS, *tef*1-α, and *tub*2 sequence information for *Pestalotiopsis* species is crucial for enabling a more robust discussion of their taxonomic status through phylogenetic analyses. Considering this issue, we conducted phylogenetic analyses using ITS, *tef*1-α, and *tub*2 sequences, and our newly obtained 21 strains nested within the genus *Pestalotiopsis* formed six independent lineages with reliable support values and can be proposed as five new phylogenetic species, namely *P. acericola*, *P. corchorifolii*, *P. fraseri*, *P. goeppertiae*, and *P. koelreuteriae*, and one known species, *P. machiliana*.

Appendages are often used as an important basis for the morphological identification of *Pestalotiopsis* species, but studies have confirmed their critical roles in conidial dispersal and host colonization (Nag Raj 1993; Gareth Jones 2006; Crous et al. 2012). This facilitates the widespread colonization of various plants by *Pestalotiopsis* as endophytes and pathogens, thereby enhancing host diversity and competitiveness for ecological niches. *Pestalotiopsis* causes diseases on a variety of host plants as a pathogen, including tea, ericaceous plants, grapes, pomegranates, blueberries, and other crops, resulting in significant economic losses (Horikawa 1986; Xu et al. 1999; McQuilken and Hopkins 2004; Keith et al. 2006; Espinoza et al. 2008; Joshi et al. 2009). Many *Pestalotiopsis* species, when living as endophytes, have been demonstrated to produce numerous secondary metabolites with diverse structural characteristics (Xu et al. 2010), and previous studies have reported over 160 different compounds isolated from *Pestalotiopsis* species, which demonstrate significant potential in antifungal and antimicrobial activities and compound synthesis (Xu et al. 2014). Thus, further research is necessary to contribute to the fields of plant pathology and fungal taxonomy and to explore their functional roles.

## Supplementary Material

XML Treatment for Pestalotiopsis acericola

XML Treatment for Pestalotiopsis corchorifolii

XML Treatment for Pestalotiopsis fraseri

XML Treatment for Pestalotiopsis goeppertiae

XML Treatment for Pestalotiopsis koelreuteriae

XML Treatment for Pestalotiopsis machiliana

## Additional information

### Use of AI

No use of AI was reported.

### Author ORCIDs

Ming-Gen Liao https://orcid.org/0009-0001-9537-1773

Zhao-Huan Xu https://orcid.org/0009-0008-2641-7783

Xiu-Guo Zhang https://orcid.org/0000-0001-9733-8494

Jian Ma https://orcid.org/0000-0001-9783-1860

### Data availability

All of the data that support the findings of this study are available in the main text or Supplementary Information.

## Supplementary materials

Supplementary material 1 Supplementary file S1, file S2 and file S3 Data type zip Explanation note **file S1**. The concatenated ITS, tef1-α and tub2 sequences; **file S2**. The sequence alignment based on ITS, tef1-α and tub2 regions, respectively; **file S3**. The PTP analysis for the Pestalotiopsis species complex and related taxa. https://binary.pensoft.net/file/1538280This dataset is made available under the Open Database License (http://opendatacommons.org/licenses/odbl/1.0/). The Open Database License (ODbL) is a license agreement intended to allow users to freely share, modify, and use this Dataset while maintaining this same freedom for others, provided that the original source and author(s) are credited. Ming-Gen Liao, Fang-Hua Guo, Xing-Xing Luo, Lian-Hu Zhang, Ru-Qiang Cui, Zhao-Huan Xu, Xiu-Guo Zhang, Jian Ma
