# Supplementary material for: Morpho-phylogenetic evidence reveals five novel species of Pestalotiopsis (Sporocadaceae, Amphisphaeriales) from southern China
Source: MycoKeys. 2026 Feb 11;128:197–230. doi: 10.3897/mycokeys.128.181974 (PMC12917497; doi:10.3897/mycokeys.128.181974)
Supplement: Supplementary material 1 — Supplementary file S1, file S2 and file S3 [file mycokeys-128-197-s001.zip › Supplementary File S1, File S2 and File S3/Supplementary File S2_The sequence alignment based on ITS, tef1-α and tub2 regions, respectively.pdf]

Supplementary File S2_The sequence alignment based on ITS, *tef1-α* and *tub2* regions, respectively.

1) The sequence alignment of *P. acericola* (HJAUP C1856.1[T]) and *P. ficicrescens* (HGUP 861[T]).

① based on ITS:

Score	Expect	Identities	Gaps	Strand
983 bits(532)	0.0	537/539(99%)	2/539(0%)	Plus/Plus

```
Query  61   CATTATAGAGTTTTCTAAACTCCCAACCCATGTGAACTTACCATTGTTGCCTCGGCAGAA  120
            ||||||||||||||||||||||||||||||||||||||||||||||||||||||||||||
Sbjct  1    CATTATAGAGTTTTCTAAACTCCCAACCCATGTGAACTTACCATTGTTGCCTCGGCAGAA  60

Query  121  GCTGCTCGGTACACCTTACCTTGGAACGGCCTACCCTGTAGCGCCTTACCCTGGAACGGC  180
            ||||||||||||||||||||||||||||||||||||||||||||||||||||||||||||
Sbjct  61   GCTGCTCGGTACACCTTACCTTGGAACGGCCTACCCTGTAGCGCCTTACCCTGGAACGGC  120

Query  181  TTACCCTGTAACGGCTGCCGGTGGACTACCAAACTCTTGTTATTTTATTGTAATCTGAGC  240
            ||||||||||||||||||||||||||||||||||||||||||||||||||||||||||||
Sbjct  121  TTACCCTGTAACGGCTGCCGGTGGACTACCAAACTCTTGTTATTTTATTGTAATCTGAGC  180

Query  241  GTCTTATTTTAATAAGTCAAAACTTTCAACAACGGATCTCTTGGTTCTGGCATCGATGAA  300
            ||||||||||||||||||||||||||||||||||||||||||||||||||||||||||||
Sbjct  181  GTCTTATTTTAATAAGTCAAAACTTTCAACAACGGATCTCTTGGTTCTGGCATCGATGAA  240

Query  301  GAACGCAGCGAAATGCGATAAGTAATGTGAATTGCAGAATTCAGTGAATCATCGAATCTT  360
            ||||||||||||||||||||||||||||||||||||||||||||||||||||||||||||
Sbjct  241  GAACGCAGCGAAATGCGATAAGTAATGTGAATTGCAGAATTCAGTGAATCATCGAATCTT  300

Query  361  TGAACGCACATTGCGCCCATTAGTATTCTAGTGGGCATGCCTGTTCGAGCGTCATTTCAA  420
            ||||||||||||||||||||||||||||||||||||||||||||||||||||||||||||
Sbjct  301  TGAACGCACATTGCGCCCATTAGTATTCTAGTGGGCATGCCTGTTCGAGCGTCATTTCAA  360

Query  421  CCCTTAAGCCTAGCTTAGTGTTGGGAGCCTACTGCTTTTACTAGCTGTAGCTCCTGAAAT  480
            ||||||||||||||||||||||||||||||||||||||||||||||||||||||||||||
Sbjct  361  CCCTTAAGCCTAGCTTAGTGTTGGGAGCCTACTGCTTTTACTAGCTGTAGCTCCTGAAAT  420

Query  481  ACAACGGCGGATCTGCGATATCCTCTGAGCGTAGTAATTTTTATCTCGCTTTTGACTGGA  540
            ||||||||||||||||||||||||||||||||||||||||||||||||||||||||||||
Sbjct  421  ACAACGGCGGATCTGCGATATCCTCTGAGCGTAGTAATTTTTATCTCGCTTTTGACTGGA  480

Query  541  GTTGCAGCGTCTTTAGCCGCTAAATCCCCCAATTTTTAATGGTTGACCTCGGATCAGGT  599
            ||||||||||||||||||||||||||||||||||||||||||| ||||||| |||||||
Sbjct  481  GTTGCAGCGTCTTTAGCCGCTAAATCCCCCAATTTTTAATGGT-GACCTCG-ATCAGGT  537
```

②based on *tef1-α*:

Score	Expect	Identities	Gaps	Strand
534 bits(289)	1e-156	291/292(99%)	0/292(0%)	Plus/Plus

```
Query  7    CATCGAGAAGTTCGAGAAGGTTAGTCATCCTCGCAATCCCATCATCCCCATCCTCATCAA  66
            ||||||||||||||||||||||||||||||||||||||||||||||||||||||||||||
Sbjct  1    CATCGAGAAGTTCGAGAAGGTTAGTCATCCTCGCAATCCCATCATCCCCATCCTCATCAA  60

Query  67   CATCACCTCGCAAACATTTCCACACCGGTGCCGAAAATCTGGTTTCCGCACCTGCCCATT  126
            ||||||||||||||||||||||||||||||||||||||||||||||||||||||||||||
Sbjct  61   CATCACCTCGCAAACATTTCCACACCGGTGCCGAAAATCTGGTTTCCGCACCTGCCCATT  120

Query  127  TTCCCAGACACTTACCCCGCCGCACGACCCCGCGGTGCAAACGAAAAATTTCTTATCGCA  186
            ||||||||||||||||||||||||||||||||||||||||||||||||||||||||||||
Sbjct  121  TTCCCAGACACTTACCCCGCCGCACGACCCCGCGGTGCAAACGAAAAATTTCTTATCGCA  180

Query  187  GCCCCACATCACAAACGTTTTGGCAGCCACGCACTTTGCATGACCCACAATGAACAATTG  246
            ||||||||||||||||||||||||||||||||||||||||||||||||||||||||||||
Sbjct  181  GCCCCACATCACAAACGTTTTGGCAGCCACGCACTTTGCATGACCCACAATGAACAATTG  240

Query  247  CTGACCCCGCCAAATAGGAAGCCGCCGAGCTCGGTAAGGGTTCCTTCAAGTA  298
            |||||||||||||||||||||||||||||||||| |||||||||||||||||
Sbjct  241  CTGACCCCGCCAAATAGGAAGCCGCCGAGCTCGGAAAGGGTTCCTTCAAGTA  292
```

③based on *tub2*:

Score	Expect	Identities	Gaps	Strand
817 bits(442)	0.0	451/455(99%)	1/455(0%)	Plus/Plus

```
Query  12   GGTAACCCAAATCGGTGCTGCTTTCTGGTATGTAGCCCATCTATCTCGACGCGCCTCAAC  71
            ||||| |||||| |||||||| ||||||||||||||||||||||||||||||||||||||
Sbjct  313  GGTAA-CCAAATTGGTGCTGCCTTCTGGTATGTAGCCCATCTATCTCGACGCGCCTCAAC  371

Query  72   ACGACACCCCCGGCAACTCGACAACGACATTCTCAACTGCTTGTTTGGAACCATACGAAA  131
            ||||||||||||||||||||||||||||||||||||||||||||||||||||||||||||
Sbjct  372  ACGACACCCCCGGCAACTCGACAACGACATTCTCAACTGCTTGTTTGGAACCATACGAAA  431

Query  132  GACTTGATACTGACCGGTCTATGATAGGCAAACCATCTCTGGCGAGCACGGTCTCGACAG  191
            ||||||||||||||||||||||||||||||||||||||||||||||||||||||||||||
Sbjct  432  GACTTGATACTGACCGGTCTATGATAGGCAAACCATCTCTGGCGAGCACGGTCTCGACAG  491

Query  192  CAATGGAGTGTACGTACCCTTTCCCTTGCTACTCGCTTTCTCGTGAACATGTCAGCTAAC  251
            ||||||||||||||| ||||||||||||||||||||||||||||||||||||||||||||
Sbjct  492  CAATGGAGTGTACGTCCCCTTTCCCTTGCTACTCGCTTTCTCGTGAACATGTCAGCTAAC  551

Query  252  AGTCGTGCTTGTTCAGCTACAACGGTACCTCCGAGCTCCAGCTCGAGCGTATGAGCGTCT  311
            ||||||||||||||||||||||||||||||||||||||||||||||||||||||||||||
Sbjct  552  AGTCGTGCTTGTTCAGCTACAACGGTACCTCCGAGCTCCAGCTCGAGCGTATGAGCGTCT  611

Query  312  ACTTCAACGAGGCTTCCGGCAACAAGTACGTTCCTCGTGCCGTCCTCGTCGATCTCGAGC  371
            ||||||||||||||||||||||||||||||||||||||||||||||||||||||||||||
Sbjct  612  ACTTCAACGAGGCTTCCGGCAACAAGTACGTTCCTCGTGCCGTCCTCGTCGATCTCGAGC  671

Query  372  CCGGTACCATGGATGCCGTCCGCGCCGGTCCTTTCGGTCAGCTCTTCCGCCCTGACAACT  431
            ||||||||||||||||||||||||||||||||||||||||||||||||||||||||||||
Sbjct  672  CCGGTACCATGGATGCCGTCCGCGCCGGTCCTTTCGGTCAGCTCTTCCGCCCTGACAACT  731

Query  432  TCGTCTTCGGTCAGTCCGGTGCCGGAAACAACTGG  466
            |||||||||||||||||||||||||||||||||||
Sbjct  732  TCGTCTTCGGTCAGTCCGGTGCCGGAAACAACTGG  766
```

2) The sequence alignment of *P. corchorifolii* (HJAUP C1891.1[T]) and *P. camelliicola* (HJAUP C1804.221[T]).

① based on ITS:

Score	Expect	Identities	Gaps	Strand
1158 bits(627)	0.0	630/631(99%)	1/631(0%)	Plus/Plus

```
Query  17   TGGAAGTAAAAGTCGTAACAAGGTCTCCGTTGGTGAACCAGCGGAGGGATCATTATAGAG  76
            ||||||||||||||||||||||||||||||||||||||||||||||||||||||||||||
Sbjct  15   TGGAAGTAAAAGTCGTAACAAGGTCTCCGTTGGTGAACCAGCGGAGGGATCATTATAGAG  74

Query  77   TTTTCTAAACTCCCAACCCATGTGAACTTACCATTGTTGCCTCGGCAGAAGCTACCCGGT  136
            ||||||||||||||||||||||||||||||||||||||||||||||||||||||||||||
Sbjct  75   TTTTCTAAACTCCCAACCCATGTGAACTTACCATTGTTGCCTCGGCAGAAGCTACCCGGT  134

Query  137  ACCTTACCTTGGAACGGCCTACCCTGTAGCGCCTTACCCTGGAACGGCTTACCCTGTAAC  196
            ||||||||||||||||||||||||||||||||||||||||||||||||||||||||||||
Sbjct  135  ACCTTACCTTGGAACGGCCTACCCTGTAGCGCCTTACCCTGGAACGGCTTACCCTGTAAC  194

Query  197  GGCTGCCGGTGGACTACCAAACTCTTGTTATTTTATTGTTATCTGAGCGTCTTATTTTAA  256
            ||||||||||||||||||||||||||||||||||||||||||||||||||||||||||||
Sbjct  195  GGCTGCCGGTGGACTACCAAACTCTTGTTATTTTATTGTTATCTGAGCGTCTTATTTTAA  254

Query  257  TAAGTCAAAACTTTCAACAACGGATCTCTTGGTTCTGGCATCGATGAAGAACGCAGCGAA  316
            ||||||||||||||||||||||||||||||||||||||||||||||||||||||||||||
Sbjct  255  TAAGTCAAAACTTTCAACAACGGATCTCTTGGTTCTGGCATCGATGAAGAACGCAGCGAA  314

Query  317  ATGCGATAAGTAATGTGAATTGCAGAATTCAGTGAATCATCGAATCTTTGAACGCACATT  376
            ||||||||||||||||||||||||||||||||||||||||||||||||||||||||||||
Sbjct  315  ATGCGATAAGTAATGTGAATTGCAGAATTCAGTGAATCATCGAATCTTTGAACGCACATT  374

Query  377  GCGCCCATTAGTATTCTAGTGGGCATGCCTGTTCGAGCGTCATTTCAACCCTTAAGCCTA  436
            ||||||||||||||||||||||||||||||||||||||||||||||||||||||||||||
Sbjct  375  GCGCCCATTAGTATTCTAGTGGGCATGCCTGTTCGAGCGTCATTTCAACCCTTAAGCCTA  434

Query  437  GCTTAGTGTTGGGAGCCTACTGCTTTTGCTAGCTGTAGCTCCTGAAATACAACGGCGGAT  496
            ||||||||||||||||||||||||||||||||||||||||||||||||||||||||||||
Sbjct  435  GCTTAGTGTTGGGAGCCTACTGCTTTTGCTAGCTGTAGCTCCTGAAATACAACGGCGGAT  494

Query  497  CTGCGATATCCTCTGAGCGTAGTAAttttttttCTCGCTTTTGACTGGAGTTGCAGCGTC  556
            ||||||||||||||||||||||||||||||||||||||||||||||||||||||||||||
Sbjct  495  CTGCGATATCCTCTGAGCGTAGTAATTTTTTTTCTCGCTTTTGACTGGAGTTGCAGCGTC  554

Query  557  TTTAGCCGCTAAATCCCCCAATTTTTAATGGTTGACCTCGGATCAGGTAGGAATACCCGC  616
            ||||||||||||||||||||||||||||||||||||||||||||||||||||||||||||
Sbjct  555  TTTAGCCGCTAAATCCCCCAATTTTTAATGGTTGACCTCGGATCAGGTAGGAATACCCGC  614

Query  617  TGAACTTAAGCATATCAATAAGC-GGAGGAA  646
            ||||||||||||||||||||||| |||||||
Sbjct  615  TGAACTTAAGCATATCAATAAGCCGGAGGAA  645
```

②based on *tef1-α*:

Score	Expect	Identities	Gaps	Strand
525 bits(284)	5e-154	296/301(98%)	3/301(0%)	Plus/Plus

```
Query  1    TCATCGAGAAGTTCGAGAAGGTTAGTCATCCTCGCAATCTCATC---CTCACCCCcatca  57
            ||||||||||||||||||||||||||||||||||||||||||||   |||||||||||||
Sbjct  8    TCATCGAGAAGTTCGAGAAGGTTAGTCATCCTCGCAATCTCATCATGCTCACCCCCATCA  67

Query  58   tcatcatcatcaCCTCGCAAACATCTTCAACCTGGTGCCGAAAATCTGTTTTCGCACCTG  117
            ||||||||||||||||||||||||||||||| ||||||||||||||||||||||||||||
Sbjct  68   TCATCATCATCACCTCGCAAACATCTTCAACTTGGTGCCGAAAATCTGTTTTCGCACCTG  127

Query  118  CCCATTTTCCCAGACACTTACCCCGCCGCACGACCCCGCGGTGCAAACGAAAAAATTCTT  177
            ||||| ||||||||||||||||||||||||||||||||||||||||||||||||||||||
Sbjct  128  CCCATGTTCCCAGACACTTACCCCGCCGCACGACCCCGCGGTGCAAACGAAAAAATTCTT  187

Query  178  ATCACAGCCCCACATCGCACAAACATTTTTGGCAGCCATGCACTTTTCATGACCCACATT  237
            ||||||||||||||||||||||||||||||||||||||||||||||||||||||||||||
Sbjct  188  ATCACAGCCCCACATCGCACAAACATTTTTGGCAGCCATGCACTTTTCATGACCCACATT  247

Query  238  GAGCATTTGCTGACCCCGCCAAATAGGAAGCCGCCGAGCTCGGTAAGGGTTCCTTCAAGT  297
            ||||||||||||||||||||||||||||||||||||||||||||||||||||||||||||
Sbjct  248  GAGCATTTGCTGACCCCGCCAAATAGGAAGCCGCCGAGCTCGGTAAGGGTTCCTTCAAGT  307

Query  298  A  298
            |
Sbjct  308  A  308
```

③based on *tub2*:

Score	Expect	Identities	Gaps	Strand
859 bits(465)	0.0	476/481(99%)	1/481(0%)	Plus/Plus

```
Query  13   GGTAACCAAAATCGGTGCTGCTTTCTGGTATGTAGCCCATCTACCTCGACACGCCTCAAT  72
            ||||||| ||||||||||||||||||||||||||||||||||||||||||||||||||||
Sbjct  1    GGTAACC-AAATCGGTGCTGCTTTCTGGTATGTAGCCCATCTACCTCGACACGCCTCAAT  59

Query  73   ACGACGCCTCCCGCAGCTCGACCACGACGGCCTCAACTATTTGGTTGGAACCAAACAAAA  132
            ||||||||||||||||||||||||||||||||||||||| ||||||||||||||||||||
Sbjct  60   ACGACGCCTCCCGCAGCTCGACCACGACGGCCTCAACTACTTGGTTGGAACCAAACAAAA  119

Query  133  GACTTGATACTGACCGGTCTCTGATAGGCAAACCATCTCTGGCGAGCACGGTCTCGACAG  192
            ||||||||||||||||||||||||||||||||||||||||||||||||||||||||||||
Sbjct  120  GACTTGATACTGACCGGTCTCTGATAGGCAAACCATCTCTGGCGAGCACGGTCTCGACAG  179

Query  193  CAATGGAGTGTACGTACCCTTGCCTTCTCCATTTGCCTTCCCACGAACATGTTAGCTAAC  252
            ||||||||||||||||||||||||||||| ||||||||||||||||||||||||||||||
Sbjct  180  CAATGGAGTGTACGTACCCTTGCCTTCTCTATTTGCCTTCCCACGAACATGTTAGCTAAC  239

Query  253  ACTCGTGCTTGCTCAGCTACAACGGTACCTCCGAGCTCCAGCTCGAGCGCATGAGCGTCT  312
            ||||||||||||||||||||||||||||||||||||||||||||||||||||||||||||
Sbjct  240  ACTCGTGCTTGCTCAGCTACAACGGTACCTCCGAGCTCCAGCTCGAGCGCATGAGCGTCT  299

Query  313  ACTTCAACGAGGCTTCCGGCAACAAGTACGTTCCTCGTGCCGTCCTCGTCGATCTCGAGC  372
            ||||||||||||||||||||||||||||||||||||||||||||||||||||||||||||
Sbjct  300  ACTTCAACGAGGCTTCCGGCAACAAGTACGTTCCTCGTGCCGTCCTCGTCGATCTCGAGC  359

Query  373  CCGGTACCATGGATGCCGTCCGCGCCGGTCCTTTCGGTCAGCTCTTCCGCCCTGACAACT  432
            ||||||||||||||||||||||||||||||||||||||||||||||||||||||||||||
Sbjct  360  CCGGTACCATGGATGCCGTCCGCGCCGGTCCTTTCGGTCAGCTCTTCCGCCCTGACAACT  419

Query  433  TCGTCTTCGGTCAATCCGGTGCCGGAAACAACTGGGCCAAGGGTCACTACCCTGAGGGTA  492
            ||||||||||||| |||||||||||||||||||||||||||||||||||| |||||||||
Sbjct  420  TCGTCTTCGGTCAGTCCGGTGCCGGAAACAACTGGGCCAAGGGTCACTACACTGAGGGTA  479

Query  493  A  493
            |
Sbjct  480  A  480
```

3) The sequence alignment of *P. fraseri* (HJAUP C1693.1[T]**) and *P. trachycarpicola* (OP068**[T]**).**

① based on ITS:

Range 1: 1 to 540 Graphics ▼ Next Match ▲ Previous Match

Score	Expect	Identities	Gaps	Strand
941 bits(509)	0.0	530/540(98%)	1/540(0%)	Plus/Plus

```
Query  50   CATTATAGAGTTTTCTAAACTCCCAACCCATGTGAACTTACCATTGTTGCCTCGGCAGAA  109
            |||||| |||||||||||||||||||||||||||||||||||||||||||||||||||||
Sbjct  1    CATTATGGAGTTTTCTAAACTCCCAACCCATGTGAACTTACCATTGTTGCCTCGGCAGAA  60

Query  110  GCTGCTCGGTGCACCCTACCTTGGAACGGCCTACCCTGTAGCGCCTTACCCTGGAACGGC  169
            ||||||||||||||||||||||||||||||||||||||||||||||||||||||||||||
Sbjct  61   GCTGCTCGGTGCACCCTACCTTGGAACGGCCTACCCTGTAGCGCCTTACCCTGGAACGGC  120

Query  170  TTACCCTGTAACGGCTGCCGGTGGACTACCAAACTCTTGTTATTTTATTGTAATCTGAGC  229
            ||||||||||||||||||||||||||||||||||||||||||||||||||||||||||||
Sbjct  121  TTACCCTGTAACGGCTGCCGGTGGACTACCAAACTCTTGTTATTTTATTGTAATCTGAGC  180

Query  230  GTCTTATTTTAATAAGTCAAAACTTTCAACAACGGATCTCTTGGTTCTGGCATCGATGAA  289
            ||||||||||||||||||||||||||||||||||||||||||||||||||||||||||||
Sbjct  181  GTCTTATTTTAATAAGTCAAAACTTTCAACAACGGATCTCTTGGTTCTGGCATCGATGAA  240

Query  290  GAACGCAGCGAAATGCGATAAGTAATGTGAATTGCATAATTCAGTGAATCATCTAATCTT  349
            |||||||||||||||||||||||||||||||||||| |||||||||||||||| ||||||
Sbjct  241  GAACGCAGCGAAATGCGATAAGTAATGTGAATTGCAGAATTCAGTGAATCATCGAATCTT  300

Query  350  TGAACGCACATTGCGCCCATTACTATTCTAGCGGGCATGCCTGTTCGAGCGTCATTTCAA  409
            |||||||||||||||||||||| |||||||| ||||||||||||||||||||||||||||
Sbjct  301  TGAACGCACATTGCGCCCATTAGTATTCTAGTGGGCATGCCTGTTCGAGCGTCATTTCAA  360

Query  410  CCCTTAAGCCTAGCTTT-TGTTGGGAGCCTACTGCTTTTGCTAGCTGTATCTCCTGAAAT  468
            ||||||||||||||||  ||||||||||||||||||||||||||||||| ||||||||||
Sbjct  361  CCCTTAAGCCTAGCTTAGTGTTGGGAGCCTACTGCTTTTGCTAGCTGTAGCTCCTGAAAT  420

Query  469  ACAACGGCGGATCTGCGATATCCTCTGAGCGTACTAAATTTTTATCTCGCTTTTGACTGG  528
            ||||||||||||||||||||||||||||||||| ||||||||||||||||||||||||||
Sbjct  421  ACAACGGCGGATCTGCGATATCCTCTGAGCGTAGTAAATTTTTATCTCGCTTTTGACTGG  480

Query  529  AGTTGCACCGTCTTTGGCCGCTAAATCCCCCAATTTTTAATGGTTGACCTCGGATCAGGT  588
            ||||||| ||||||||||||||||||||||||||||||||||||||||||||||||||||
Sbjct  481  AGTTGCAGCGTCTTTGGCCGCTAAATCCCCCAATTTTTAATGGTTGACCTCGGATCAGGT  540
```

②based on *tef1-α*:

Range 1: 37 to 331 Graphics ▼ Next Match ▲ Previous Match

Score	Expect	Identities	Gaps	Strand
540 bits(292)	6e-158	294/295(99%)	0/295(0%)	Plus/Plus

```
Query  2    CATCGAGAAGTTCGAGAAGGTTAGtcatcctcgcaatcccatcatcctcatcctcatcat  61
            ||||||||||||||||||||||||||||||||||||||||||||||||||||||||||||
Sbjct  37   CATCGAGAAGTTCGAGAAGGTTAGTCATCCTCGCAATCCCATCATCCTCATCCTCATCAT  96

Query  62   catcaCTTCGCAAACATTTCCCACACCGGTGTCGAAAATCTGGTTTTCGCACCTGCCCAT  121
            ||||||||||||||||||||||||||||||||||||||||||||||||||||||||||||
Sbjct  97   CATCACTTCGCAAACATTTCCCACACCGGTGTCGAAAATCTGGTTTTCGCACCTGCCCAT  156

Query  122  TTTCTCAGACACTTACCCCGCCGCACGACCCCGCGGTGCAAACGAAAAATTTCTTATCAC  181
            ||||||||||||||||||||||||||||||||||||||||||||||||||||||||||||
Sbjct  157  TTTCTCAGACACTTACCCCGCCGCACGACCCCGCGGTGCAAACGAAAAATTTCTTATCAC  216

Query  182  AGCCCCACATCACACAAACATTTTGGCAGCCACGCACCTTGCATGACCCACAATGAACAA  241
            ||||||||||||||||||||||||||||||||||||||||||||||||||||||||||||
Sbjct  217  AGCCCCACATCACACAAACATTTTGGCAGCCACGCACCTTGCATGACCCACAATGAACAA  276

Query  242  TTGCTGACCCCGCCAAATAGGAAGCCGCCGAGCTCGGTAAGGGTTCCTTCAAGTA  296
            ||||||||||||||||||||||||||||||||||||| |||||||||||||||||
Sbjct  277  TTGCTGACCCCGCCAAATAGGAAGCCGCCGAGCTCGGAAAGGGTTCCTTCAAGTA  331
```

③based on *tub2*:

```
Range 1: 1 to 453 Graphics                                   ▼ Next Match ▲ Previous Match

Score          Expect   Identities       Gaps       Strand
837 bits(453)  0.0      453/453(100%)    0/453(0%)  Plus/Plus

Query  24   CGGTGCTGCTTTCTGGTATGTAGCCCATCTACCTCGACGCGCCTCAATACGACACCCCCG  83
            ||||||||||||||||||||||||||||||||||||||||||||||||||||||||||||
Sbjct  1    CGGTGCTGCTTTCTGGTATGTAGCCCATCTACCTCGACGCGCCTCAATACGACACCCCCG  60

Query  84   GCAACTCGACCACGATAATCTCAACTGCTTGGTTGGAACCATACGAAAGACTCGATACTG  143
            ||||||||||||||||||||||||||||||||||||||||||||||||||||||||||||
Sbjct  61   GCAACTCGACCACGATAATCTCAACTGCTTGGTTGGAACCATACGAAAGACTCGATACTG  120

Query  144  ACCGGTCTATGATAGGCAAACCATCTCTGGCGAGCACGGTCTCGACAGCAATGGAGTGTA  203
            ||||||||||||||||||||||||||||||||||||||||||||||||||||||||||||
Sbjct  121  ACCGGTCTATGATAGGCAAACCATCTCTGGCGAGCACGGTCTCGACAGCAATGGAGTGTA  180

Query  204  CGTACCCTTTCCCTGGCTGTTCGCTTTCTCGTGAACATGTCAGCTAACAGTCGTGCTTGT  263
            ||||||||||||||||||||||||||||||||||||||||||||||||||||||||||||
Sbjct  181  CGTACCCTTTCCCTGGCTGTTCGCTTTCTCGTGAACATGTCAGCTAACAGTCGTGCTTGT  240

Query  264  TCAGCTACAACGGTACCTCCGAGCTCCAGCTCGAGCGCATGAGCGTCTACTTCAACGAGG  323
            ||||||||||||||||||||||||||||||||||||||||||||||||||||||||||||
Sbjct  241  TCAGCTACAACGGTACCTCCGAGCTCCAGCTCGAGCGCATGAGCGTCTACTTCAACGAGG  300

Query  324  CTTCCGGCAACAAGTACGTTCCTCGTGCCGTCCTCGTCGATCTCGAGCCCGGTACCATGG  383
            ||||||||||||||||||||||||||||||||||||||||||||||||||||||||||||
Sbjct  301  CTTCCGGCAACAAGTACGTTCCTCGTGCCGTCCTCGTCGATCTCGAGCCCGGTACCATGG  360

Query  384  ATGCCGTCCGCGCCGGTCCTTTCGGTCAGCTCTTCCGCCCTGACAACTTCGTCTTCGGTC  443
            ||||||||||||||||||||||||||||||||||||||||||||||||||||||||||||
Sbjct  361  ATGCCGTCCGCGCCGGTCCTTTCGGTCAGCTCTTCCGCCCTGACAACTTCGTCTTCGGTC  420

Query  444  AGTCCGGTGCCGGAAACAACTGGGCCAAGGGTC  476
            |||||||||||||||||||||||||||||||||
Sbjct  421  AGTCCGGTGCCGGAAACAACTGGGCCAAGGGTC  453
```

4) The sequence alignment of *P. goeppertiae* (HJAUP C1919.1^{T}) and *P. neolitseae* (NTUCC 17–011^{T}).

① based on ITS:

```
Score          Expect   Identities       Gaps       Strand
966 bits(523)  0.0      523/523(100%)    0/523(0%)  Plus/Plus

Query  53   CATTATAGAGTTTTCTAAACTCCCAACCCATGTGAACTTACCATTGTTGCCTCGGCAGAA  112
            ||||||||||||||||||||||||||||||||||||||||||||||||||||||||||||
Sbjct  1    CATTATAGAGTTTTCTAAACTCCCAACCCATGTGAACTTACCATTGTTGCCTCGGCAGAA  60

Query  113  GCTGCTCGGCGCGCCTTACCTTGGAACGGCCTACCCTGTAGCGCCTTACCCTGGAACGGC  172
            ||||||||||||||||||||||||||||||||||||||||||||||||||||||||||||
Sbjct  61   GCTGCTCGGCGCGCCTTACCTTGGAACGGCCTACCCTGTAGCGCCTTACCCTGGAACGGC  120

Query  173  TTACCCTGCAACGGCTGCCGGTGGACTACCAAACTCTTGTTATTTTATGGTTATCTGAGC  232
            ||||||||||||||||||||||||||||||||||||||||||||||||||||||||||||
Sbjct  121  TTACCCTGCAACGGCTGCCGGTGGACTACCAAACTCTTGTTATTTTATGGTTATCTGAGC  180

Query  233  GTCTTATTTTAATAAGTCAAAACTTTCAACAACGGATCTCTTGGTTCTGGCATCGATGAA  292
            ||||||||||||||||||||||||||||||||||||||||||||||||||||||||||||
Sbjct  181  GTCTTATTTTAATAAGTCAAAACTTTCAACAACGGATCTCTTGGTTCTGGCATCGATGAA  240

Query  293  GAACGCAGCGAAATGCGATAAGTAATGTGAATTGCAGAATTCAGTGAATCATCGAATCTT  352
            ||||||||||||||||||||||||||||||||||||||||||||||||||||||||||||
Sbjct  241  GAACGCAGCGAAATGCGATAAGTAATGTGAATTGCAGAATTCAGTGAATCATCGAATCTT  300

Query  353  TGAACGCACATTGCGCCCATTAGTATTCTAGTGGGCATGCCTGTTCGAGCGTCATTTCAA  412
            ||||||||||||||||||||||||||||||||||||||||||||||||||||||||||||
Sbjct  301  TGAACGCACATTGCGCCCATTAGTATTCTAGTGGGCATGCCTGTTCGAGCGTCATTTCAA  360

Query  413  CCCTTAAGCCTAGCTTAGTGTTGGGAGCCTACTGCTTTTGCTAGCTGTAGCTCCTGAAAT  472
            ||||||||||||||||||||||||||||||||||||||||||||||||||||||||||||
Sbjct  361  CCCTTAAGCCTAGCTTAGTGTTGGGAGCCTACTGCTTTTGCTAGCTGTAGCTCCTGAAAT  420

Query  473  ACAACGGCGGATCTGCGATATCCTCTGAGCGTAGTAATTTTTATCTCGCTTTTGACTGGA  532
            ||||||||||||||||||||||||||||||||||||||||||||||||||||||||||||
Sbjct  421  ACAACGGCGGATCTGCGATATCCTCTGAGCGTAGTAATTTTTATCTCGCTTTTGACTGGA  480

Query  533  GTTGCAGCGTCTTTAGCCGCTAAACCCCCCAATTTTTAATGGT  575
            |||||||||||||||||||||||||||||||||||||||||||
Sbjct  481  GTTGCAGCGTCTTTAGCCGCTAAACCCCCCAATTTTTAATGGT  523
```

②based on *tef1-α*:

```
Score           Expect    Identities      Gaps        Strand
422 bits(228)   1e-122    248/257(96%)    4/257(1%)   Plus/Plus

Query  36     CCTCGCAATCCCATCATCCCCATCCTCATACCTCGCAAACGTTTTCCAACCGGTGCCGAA  95
              || ||| || ||||||| |||||||||||||||||| |||||||||||||||||| ||||
Sbjct  2      CCCCGC-AT-CCATCAT-CCCATCCTCATACCTCGC-AACGTTTTCCAACCGGTGTCGAA  57

Query  96     AATCTGTTTTCGCACCTGCCCATTTTCCCAGACACTTACCCCGCCGCACGACCCCGCGGT  155
              |||| |||||||||||||||||||||||||||||||||||||||||||||||||||||||
Sbjct  58     AATCGGTTTTCGCACCTGCCCATTTTCCCAGACACTTACCCCGCCGCACGACCCCGCGGT  117

Query  156    GCAAACGAAAAATTTCTTATCACAGCCCCACTTCACACAACATTTTGGGAGCCACGCACT  215
              |||||||||||||||||||||||||||||||||||||||||||||||| |||||||||||
Sbjct  118    GCAAACGAAAAATTTCTTATCACAGCCCCACTTCACACAACATTTTGGCAGCCACGCACT  177

Query  216    TTGCATGACCCACAGTGAACAATTGCTGACCCCGCCAAATAGGAAGCCGCCGAGCTCGGT  275
              ||||||||||||||||||||||||||||||||||||||||||||||||||||||||||| 
Sbjct  178    TTGCATGACCCACAGTGAACAATTGCTGACCCCGCCAAATAGGAAGCCGCCGAGCTCGGA  237

Query  276    AAGGGTTCCTTCAAGTA  292
              |||||||||||||||||
Sbjct  238    AAGGGTTCCTTCAAGTA  254
```

③based on *tub2*:

```
Score           Expect    Identities      Gaps        Strand
734 bits(397)   0.0       399/400(99%)    0/400(0%)   Plus/Plus

Query  71     ATACGACACCCTCCGCAACTCGACGACGACATTCTCGGCTTCTTGGTTGGAACCGAACCA  130
              ||||||||||||||||||||||||||||||||||||||||||||||||||||||||||||
Sbjct  2      ATACGACACCCTCCGCAACTCGACGACGACATTCTCGGCTTCTTGGTTGGAACCGAACCA  61

Query  131    AAGACTTGATACTGACCGGTCTCTGATAGGCAAACCATCTCTGGCGAGCACGGTCTCGAC  190
              ||||||||||||||||||||||||||||||||||||||||||||||||||||||||||||
Sbjct  62     AAGACTTGATACTGACCGGTCTCTGATAGGCAAACCATCTCTGGCGAGCACGGTCTCGAC  121

Query  191    AGCAATGGAGTGTACGTACCGTTTCCTTGCCTACTTGCTTTCCCACGAACATGTTAGCTA  250
              ||||||||||||||||||||||| ||||||||||||||||||||||||||||||||||||
Sbjct  122    AGCAATGGAGTGTACGTACCGTTCCCTTGCCTACTTGCTTTCCCACGAACATGTTAGCTA  181

Query  251    ACACTCGTGCTTGCTCAGCTACAACGGCACCTCCGAGCTCCAGCTCGAGCGCATGAGCGT  310
              ||||||||||||||||||||||||||||||||||||||||||||||||||||||||||||
Sbjct  182    ACACTCGTGCTTGCTCAGCTACAACGGCACCTCCGAGCTCCAGCTCGAGCGCATGAGCGT  241

Query  311    CTACTTCAACGAGGCTTCCGGCAACAAGTACGTTCCTCGTGCCGTCCTCGTCGATCTCGA  370
              ||||||||||||||||||||||||||||||||||||||||||||||||||||||||||||
Sbjct  242    CTACTTCAACGAGGCTTCCGGCAACAAGTACGTTCCTCGTGCCGTCCTCGTCGATCTCGA  301

Query  371    GCCCGGTACCATGGACGCCGTCCGCGCCGGTCCTTTCGGCCAGCTCTTCCGCCCTGACAA  430
              ||||||||||||||||||||||||||||||||||||||||||||||||||||||||||||
Sbjct  302    GCCCGGTACCATGGACGCCGTCCGCGCCGGTCCTTTCGGCCAGCTCTTCCGCCCTGACAA  361

Query  431    CTTCGTCTTCGGTCAGTCCGGTGCCGGAAACAACTGGGCC  470
              ||||||||||||||||||||||||||||||||||||||||
Sbjct  362    CTTCGTCTTCGGTCAGTCCGGTGCCGGAAACAACTGGGCC  401
```

5) The sequence alignment of *P. fraseri* (HJAUP C1693.1T) and *P. koelreuteriae* (HJAUP C2757.1T).

① based on ITS:

Score	Expect	Identities	Gaps	Strand
1092 bits(591)	0.0	619/632(98%)	3/632(0%)	Plus/Plus

```
Query  1    GGAAGTAAAAA-TCGTAACAAGGTCTCCGTTGGTGAACCAGCGGAGGGATCATTATAGAG  59
            ||||||||||| ||||||||||||||||||||||||||||||||||||||||||||||||
Sbjct  3    GGAAGTAAAAAGTCGTAACAAGGTCTCCGTTGGTGAACCAGCGGAGGGATCATTATAGAG  62

Query  60   TTTTCTAAACTCCCAACCCATGTGAACTTACCATTGTTGCCTCGGCAGAAGCTGCTCGGT  119
            ||||||||||||||||||||||||||||||||||||||||||||||||||||||||||||
Sbjct  63   TTTTCTAAACTCCCAACCCATGTGAACTTACCATTGTTGCCTCGGCAGAAGCTGCTCGGT  122

Query  120  GCACCCTACCTTGGAACGGCCTACCCTGTAGCGCCTTACCCTGGAACGGCTTACCCTGTA  179
            ||||||||||||||||||||||||||||||||||||||||||||||||||||||||||||
Sbjct  123  GCACCCTACCTTGGAACGGCCTACCCTGTAGCGCCTTACCCTGGAACGGCTTACCCTGTA  182

Query  180  ACGGCTGCCGGTGGACTACCAAACTCTTGTTATTTTATTGTAATCTGAGCGTCTTATTTT  239
            ||||||||||||||||||||||||||||||||||||||||||||||||||||||||||||
Sbjct  183  ACGGCTGCCGGTGGACTACCAAACTCTTGTTATTTTATTGTAATCTGAGCGTCTTATTTT  242

Query  240  AATAAGTCAAAACTTTCAACAACGGATCTCTTGGTTCTGGCATCGATGAAGAACGCAGCG  299
            ||||||||||||||||||||||||||||||||||||||||||||||||||||||||||||
Sbjct  243  AATAAGTCAAAACTTTCAACAACGGATCTCTTGGTTCTGGCATCGATGAAGAACGCAGCG  302

Query  300  AAATGCGATAAGTAATGTGAATTGCATAATTCAGTGAATCATCTAATCTTTGAACGCACA  359
            |||||||||||||||||||||||||| |||||||||||||||| ||||||||||||||||
Sbjct  303  AAATGCGATAAGTAATGTGAATTGCAGAATTCAGTGAATCATCGAATCTTTGAACGCACA  362

Query  360  TTGCGCCCATTACTATTCTAGCGGGCATGCCTGTTCGAGCGTCATTTCAACCCTTAAGCC  419
            |||||||||||| |||||||| ||||||||||||||||||||||||||||||||||||||
Sbjct  363  TTGCGCCCATTAGTATTCTAGTGGGCATGCCTGTTCGAGCGTCATTTCAACCCTTAAGCC  422

Query  420  TAGCTTT-TGTTGGGAGCCTACTGCTTTTGCTAGCTGTATCTCCTGAAATACAACGGCGG  478
            ||||||  ||||||||||||||||||||||||||||||| ||||||||||||||||||||
Sbjct  423  TAGCTTAGTGTTGGGAGCCTACTGCTTTTGCTAGCTGTAGCTCCTGAAATACAACGGCGG  482

Query  479  ATCTGCGATATCCTCTGAGCGTACTAAATTTTTATCTCGCTTTTGACTGGAGTTGCACCG  538
            ||||||||||||||||||||||| ||||||||||||||||||||||||||||||||| ||
Sbjct  483  ATCTGCGATATCCTCTGAGCGTAGTAAATTTTTATCTCGCTTTTGACTGGAGTTGCAGCG  542

Query  539  TCTTTGGCCGCTAAATCCCCCAATTTTTAATGGTTGACCTCGGATCAGGTAGGAATACCC  598
            ||||||||||||||||||||||||||||||||||||||||||||||||||||||||||||
Sbjct  543  TCTTTGGCCGCTAAATCCCCCAATTTTTAATGGTTGACCTCGGATCAGGTAGGAATACCC  602

Query  599  GCTGAACTTAATCATATCAATAA-CCGCAGGA  629
            ||||||||||| ||||||||||| ||| ||||
Sbjct  603  GCTGAACTTAAGCATATCAATAAGCCGGAGGA  634
```

②based on *tef1-α*:

Score	Expect	Identities	Gaps	Strand
544 bits(294)	1e-159	296/297(99%)	0/297(0%)	Plus/Plus

```
Query  1    TCATCGAGAAGTTCGAGAAGGTTAGtcatcctcgcaatcccatcatcctcatcctcatca  60
            ||||||||||||||||||||||||||||||||||||||||||||||||||||||||||||
Sbjct  1    TCATCGAGAAGTTCGAGAAGGTTAGTCATCCTCGCAATCCCATCATCCTCATCCTCATCA  60

Query  61   tcatcaCTTCGCAAACATTTCCCACACCGGTGTCGAAAATCTGGTTTTCGCACCTGCCCA  120
            ||||||||||||||||||||||||||||||||| ||||||||||||||||||||||||||
Sbjct  61   TCATCACTTCGCAAACATTTCCCACACCGGTGTAGAAAATCTGGTTTTCGCACCTGCCCA  120

Query  121  TTTTCTCAGACACTTACCCCGCCGCACGACCCCGCGGTGCAAACGAAAAATTTCTTATCA  180
            ||||||||||||||||||||||||||||||||||||||||||||||||||||||||||||
Sbjct  121  TTTTCTCAGACACTTACCCCGCCGCACGACCCCGCGGTGCAAACGAAAAATTTCTTATCA  180

Query  181  CAGCCCCACATCACACAAACATTTTGGCAGCCACGCACCTTGCATGACCCACAATGAACA  240
            ||||||||||||||||||||||||||||||||||||||||||||||||||||||||||||
Sbjct  181  CAGCCCCACATCACACAAACATTTTGGCAGCCACGCACCTTGCATGACCCACAATGAACA  240

Query  241  ATTGCTGACCCCGCCAAATAGGAAGCCGCCGAGCTCGGTAAGGGTTCCTTCAAGTAA  297
            |||||||||||||||||||||||||||||||||||||||||||||||||||||||||
Sbjct  241  ATTGCTGACCCCGCCAAATAGGAAGCCGCCGAGCTCGGTAAGGGTTCCTTCAAGTAA  297
```

③based on *tub2*:

```
Score            Expect   Identities      Gaps         Strand
872 bits(472)    0.0      479/482(99%)    1/482(0%)    Plus/Plus

Query  1    TTGGTAACCAAATCGGTGCTGCTTTCTGGTATGTAGCCCATCTACCTCGACGCGCCTCAA  60
            ||||||||||||||||||||||||||||||||||||||||||||||||||||||||||||
Sbjct  11   TTGGTAACCAAATCGGTGCTGCTTTCTGGTATGTAGCCCATCTACCTCGACGCGCCTCAA  70

Query  61   TACGACACCCCCGGCAACTCGACCACGATAATCTCAACTGCTTGGTTGGAACCATACGAA  120
            ||||||||||||||||||||||||||||||||||||||||||||||||||||||||||||
Sbjct  71   TACGACACCCCCGGCAACTCGACCACGATAATCTCAACTGCTTGGTTGGAACCATACGAA  130

Query  121  AGACTCGATACTGACCGGTCTATGATAGGCAAACCATCTCTGGCGAGCACGGTCTCGACA  180
            ||||||||||||||||||||||||||||||||||||||||||||||||||||||||||||
Sbjct  131  AGACTCGATACTGACCGGTCTATGATAGGCAAACCATCTCTGGCGAGCACGGTCTCGACA  190

Query  181  GCAATGGAGTGTACGTACCCTTTCCCTGGCTGTTCGCTTTCTCGTGAACATGTCAGCTAA  240
            ||||||||||||||||||||||||||||||||||||||||||||||||||||||||||||
Sbjct  191  GCAATGGAGTGTACGTACCCTTTCCCTGGCTGTTCGCTTTCTCGTGAACATGTCAGCTAA  250

Query  241  CAGTCGTGCTTGTTCAGCTACAACGGTACCTCCGAGCTCCAGCTCGAGCGCATGAGCGTC  300
            ||||||||||||||||||||||||||||||||||||||||||||||||||||||||||||
Sbjct  251  CAGTCGTGCTTGTTCAGCTACAACGGTACCTCCGAGCTCCAGCTCGAGCGCATGAGCGTC  310

Query  301  TACTTCAACGAGGCTTCCGGCAACAAGTACGTTCCTCGTGCCGTCCTCGTCGATCTCGAG  360
            ||||||||||||||||||||||||||||||||||||||||||||||||||||||||||||
Sbjct  311  TACTTCAACGAGGCTTCCGGCAACAAGTACGTTCCTCGTGCCGTCCTCGTCGATCTCGAG  370

Query  361  CCCGGTACCATGGATGCCGTCCGCGCCGGTCCTTTCGGTCAGCTCTTCCGCCCTGACAAC  420
            ||||||||||||||||||||||||||||||||||||||||||||||||||||||||||||
Sbjct  371  CCCGGTACCATGGATGCCGTCCGCGCCGGTCCTTTCGGTCAGCTCTTCCGCCCTGACAAC  430

Query  421  TTCGTCTTCGGTCAGTCCGGTGCCGGAAACAACTGGGCCAAGGGTCACTACACT-GAGGG  479
            ||||||||||||||||||||||||||||||||||||||||||||||||||  ||  |||||
Sbjct  431  TTCGTCTTCGGTCAGTCCGGTGCCGGAAACAACTGGGCCAAGGGTCACTAACCTTGAGGG  490

Query  480  TA  481
            ||
Sbjct  491  TA  492
```

6) The sequence alignment of *P. machiliana* (HJAUP C2801.1) and *P. machiliana* (HJAUP C1790.221^{T}).

① based on ITS:

```
Score            Expect   Identities      Gaps         Strand
1155 bits(625)   0.0      630/632(99%)    1/632(0%)    Plus/Plus

Query  1    TTGGAAGTAAAAGTCGTAACAAGGTCTCCGTTGGTGAACCAGCGGAGGGATCATTATAGA  60
            ||||||||||||||||||||||||||||||||||||||||||||||||||||||||||||
Sbjct  1    TTGGAAGTAAAAGTCGTAACAAGGTCTCCGTTGGTGAACCAGCGGAGGGATCATTATAGA  60

Query  61   GTTTTCTAAACTCCCAACCCATGTGAACTTACCATTGTTGCCTCGGCAGAAGCTACCTGG  120
            ||||| ||||||||||||||||||||||||||||||||||||||||||||||||||||||
Sbjct  61   GTTTTTTAAACTCCCAACCCATGTGAACTTACCATTGTTGCCTCGGCAGAAGCTACCTGG  120

Query  121  TTACCTTACCTTGGAACGGCCTACCCTGTAGCGCCTTACCCTGGAACGGCCTACCCTGTA  180
            ||||||||||||||||||||||||||||||||||||||||||||||||||||||||||||
Sbjct  121  TTACCTTACCTTGGAACGGCCTACCCTGTAGCGCCTTACCCTGGAACGGCCTACCCTGTA  180

Query  181  ACGGCTGCCGGTGGACTACCAAACTCTTGTTATTATATTGTAATCTGAGCGTCTTATTTT  240
            ||||||||||||||||||||||||||||||||||||||||||||||||||||||||||||
Sbjct  181  ACGGCTGCCGGTGGACTACCAAACTCTTGTTATTATATTGTAATCTGAGCGTCTTATTTT  240

Query  241  AATAAGTCAAAACTTTCAACAACGGATCTCTTGGTTCTGGCATCGATGAAGAACGCAGCG  300
            ||||||||||||||||||||||||||||||||||||||||||||||||||||||||||||
Sbjct  241  AATAAGTCAAAACTTTCAACAACGGATCTCTTGGTTCTGGCATCGATGAAGAACGCAGCG  300

Query  301  AAATGCGATAAGTAATGTGAATTGCAGAATTCAGTGAATCATCGAATCTTTGAACGCACA  360
            ||||||||||||||||||||||||||||||||||||||||||||||||||||||||||||
Sbjct  301  AAATGCGATAAGTAATGTGAATTGCAGAATTCAGTGAATCATCGAATCTTTGAACGCACA  360

Query  361  TTGCGCCCATTAGTATTCTAGTGGGCATGCCTGTTCGAGCGTCATTTCAACCCTTAAGCC  420
            ||||||||||||||||||||||||||||||||||||||||||||||||||||||||||||
Sbjct  361  TTGCGCCCATTAGTATTCTAGTGGGCATGCCTGTTCGAGCGTCATTTCAACCCTTAAGCC  420

Query  421  TAGCTTAGTGTTGGGAGCCTACTGCTTTTGCTAGCGGTAGCTCCTGAAATACAACGGCGG  480
            ||||||||||||||||||||||||||||||||||||||||||||||||||||||||||||
Sbjct  421  TAGCTTAGTGTTGGGAGCCTACTGCTTTTGCTAGCGGTAGCTCCTGAAATACAACGGCGG  480

Query  481  ATCTGCGATATCCTCTGAGCGTAGTAATTTTTATCTCGCTTTTGACTGGAGTTGCAGCGT  540
            ||||||||||||||||||||||||||||||||||||||||||||||||||||||||||||
Sbjct  481  ATCTGCGATATCCTCTGAGCGTAGTAATTTTTATCTCGCTTTTGACTGGAGTTGCAGCGT  540

Query  541  CTTTAGCCGCTAAACCCCCCAATTTTTAATGGTTGACCTCGGATCAGGTAGGAATACCCG  600
            ||||||||||||||||||||||||||||||||||||||||||||||||||||||||||||
Sbjct  541  CTTTAGCCGCTAAACCCCCCAATTTTTAATGGTTGACCTCGGATCAGGTAGGAATACCCG  600

Query  601  CTGAACTTAAGCATATCAATAAGCCGGAGGAA  632
            |||||||||||||||||||||||| |||||||
Sbjct  601  CTGAACTTAAGCATATCAATAAGC-GGAGGAA  631
```

②based on *tef1-α*:

Score	Expect	Identities	Gaps	Strand
540 bits(292)	2e-158	292/292(100%)	0/292(0%)	Plus/Plus

```
Query  2    CATCGAGAAGTTCGAGAAGGTTAGTCATCCTCAAATCCCATCATTCCCATCCTCATCATC  61
            ||||||||||||||||||||||||||||||||||||||||||||||||||||||||||||
Sbjct  1    CATCGAGAAGTTCGAGAAGGTTAGTCATCCTCAAATCCCATCATTCCCATCCTCATCATC  60

Query  62   ATCGCCTCGCAAACATTTTCCAACCGGTGCCGAGAATCTGTTTTCGCATCTGCCCATTTT  121
            ||||||||||||||||||||||||||||||||||||||||||||||||||||||||||||
Sbjct  61   ATCGCCTCGCAAACATTTTCCAACCGGTGCCGAGAATCTGTTTTCGCATCTGCCCATTTT  120

Query  122  CCCAGACACTTACCCCGCCGCACGACCCCGCGGTGCAAACGAAAAAATTCTTATCACAGC  181
            ||||||||||||||||||||||||||||||||||||||||||||||||||||||||||||
Sbjct  121  CCCAGACACTTACCCCGCCGCACGACCCCGCGGTGCAAACGAAAAAATTCTTATCACAGC  180

Query  182  CCCACATCGCACAAACATTTTGGCAGCCATGCACTTTCCAAGACCCACAATGAACATTTG  241
            ||||||||||||||||||||||||||||||||||||||||||||||||||||||||||||
Sbjct  181  CCCACATCGCACAAACATTTTGGCAGCCATGCACTTTCCAAGACCCACAATGAACATTTG  240

Query  242  CTGACCCCGCCAAATAGGAAGCCGCCGAGCTCGGTAAGGGTTCCTTCAAGTA  293
            ||||||||||||||||||||||||||||||||||||||||||||||||||||
Sbjct  241  CTGACCCCGCCAAATAGGAAGCCGCCGAGCTCGGTAAGGGTTCCTTCAAGTA  292
```

③based on *tub2*:

Score	Expect	Identities	Gaps	Strand
883 bits(478)	0.0	481/482(99%)	1/482(0%)	Plus/Plus

```
Query  3    GGTAACCAAATCGGTGCTGCTTTCTGGTATGTAGCCCATCTACCTCGACACGCCTCAATA  62
            ||||||||||||||||||||||||||||||||||||||||||||||||||||||||||||
Sbjct  1    GGTAACCAAATCGGTGCTGCTTTCTGGTATGTAGCCCATCTACCTCGACACGCCTCAATA  60

Query  63   CGACAACCCCCCGCAACTCGACAACGACGTTCTCAACAAGTGCTTGCTTGGAAACAAGGG  122
            |||||| |||||||||||||||||||||||||||||||||||||||||||||||||||||
Sbjct  61   CGACAA-CCCCCGCAACTCGACAACGACGTTCTCAACAAGTGCTTGCTTGGAAACAAGGG  119

Query  123  AAAGACTTGATACTGACCGGTCCCTGATAGGCAAACCATCTCTGGCGAGCACGGTCTCGA  182
            ||||||||||||||||||||||||||||||||||||||||||||||||||||||||||||
Sbjct  120  AAAGACTTGATACTGACCGGTCCCTGATAGGCAAACCATCTCTGGCGAGCACGGTCTCGA  179

Query  183  CAGCAATGGAGTGTACGTACCCTTTCCTTGGCTACTTGCTTTCCCACGAACATCTCAGCT  242
            ||||||||||||||||||||||||||||||||||||||||||||||||||||||||||||
Sbjct  180  CAGCAATGGAGTGTACGTACCCTTTCCTTGGCTACTTGCTTTCCCACGAACATCTCAGCT  239

Query  243  AACACTCGTGGTTGTTCAGCTACAACGGTACCTCCGAGCTCCAGCTCGAGCGCATGAGCG  302
            ||||||||||||||||||||||||||||||||||||||||||||||||||||||||||||
Sbjct  240  AACACTCGTGGTTGTTCAGCTACAACGGTACCTCCGAGCTCCAGCTCGAGCGCATGAGCG  299

Query  303  TCTACTTCAACGAGGCTTCCGGCAACAAGTACGTTCCTCGTGCCGTCCTCGTCGATCTCG  362
            ||||||||||||||||||||||||||||||||||||||||||||||||||||||||||||
Sbjct  300  TCTACTTCAACGAGGCTTCCGGCAACAAGTACGTTCCTCGTGCCGTCCTCGTCGATCTCG  359

Query  363  AGCCCGGTACCATGGATGCCGTCCGCGCCGGTCCTTTCGGTCAGCTCTTCCGCCCTGACA  422
            ||||||||||||||||||||||||||||||||||||||||||||||||||||||||||||
Sbjct  360  AGCCCGGTACCATGGATGCCGTCCGCGCCGGTCCTTTCGGTCAGCTCTTCCGCCCTGACA  419

Query  423  ACTTCGTCTTCGGTCAGTCCGGTGCCGGAAACAACTGGGCCAAGGGTCACTACACTGAGG  482
            ||||||||||||||||||||||||||||||||||||||||||||||||||||||||||||
Sbjct  420  ACTTCGTCTTCGGTCAGTCCGGTGCCGGAAACAACTGGGCCAAGGGTCACTACACTGAGG  479

Query  483  GT  484
            ||
Sbjct  480  GT  481
```
