# Supplementary figures and images for: Morpho-phylogenetic evidence reveals five novel species of Pestalotiopsis (Sporocadaceae, Amphisphaeriales) from southern China
Source: MycoKeys. 2026 Feb 11;128:197–230. doi: 10.3897/mycokeys.128.181974 (PMC12917497; doi:10.3897/mycokeys.128.181974)

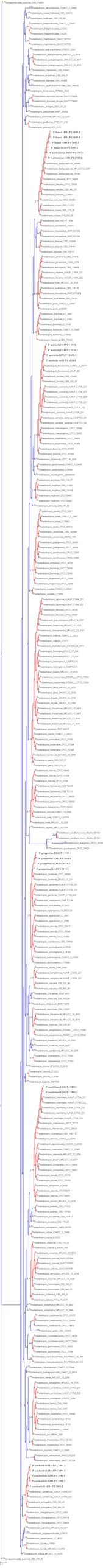

Supplement: Supplementary material 1 — Supplementary file S1, file S2 and file S3 [file mycokeys-128-197-s001.zip › Supplementary File S1, File S2 and File S3/Supplementary File S3_The PTP analysis for the Pestalotiopsis species complex and related taxa.jpg]
